# Differential Plasma Carotenoid Profiles in Hypertensive Disorders of Pregnancy

**DOI:** 10.3390/nu17193104

**Published:** 2025-09-29

**Authors:** Colman I. Freel, Jonah Scheffler, Rebecca A. Drakowski, Elizabeth Lyden, Matthew VanOrmer, Melissa K. Thoene, Paras Kumar Mishra, Corrine K. Hanson, Ann L. Anderson-Berry

**Affiliations:** 1 Department of Cellular and Integrative Physiology, University of Nebraska Medical Center, Omaha, NE 68198, USA; paraskumar.mishra@unmc.edu; 2 Division of Newborn Medicine, Department of Pediatrics, University of Nebraska Medical Center, Omaha, NE 68198, USA; jonah.scheffler@unmc.edu (J.S.); rebecca.slotkowski@unmc.edu (R.A.D.); matthew.vanormer@unmc.edu (M.V.); melissak.thoene@unmc.edu (M.K.T.); alanders@unmc.edu (A.L.A.-B.); 3 College of Public Health, University of Nebraska Medical Center, Omaha, NE 68198, USA; elyden@unmc.edu; 4 College of Allied Health Professions, University of Nebraska Medical Center, Omaha, NE 68198, USA; ckhanson@unmc.edu

**Keywords:** hypertensive disorders of pregnancy, carotenoids, antioxidants, preeclampsia, gestational hypertension, chronic hypertension, pregnancy

## Abstract

**Background**: Hypertensive disorders of pregnancy (HDP) affect one in six pregnancies globally. The etiology of HDP remains unclear but is known to involve oxidative stress. While the body produces endogenous antioxidants, antioxidative nutrients, like carotenoids, remain critical in modulating oxidative stress. The statuses of several carotenoids have been linked to hypertension in both pregnant and non-pregnant populations. However, their associations with the spectrum of HDP, including gestational hypertension (GH), chronic hypertension (CH), and preeclampsia (PE), remains unclear. Our objective was to quantify and compare carotenoid intake and plasma levels among HDP. **Methods**: We conducted a prospective cohort study of patients presenting for delivery at a Midwestern academic medical center between 2015 and 2023. Women ≥ 19 years old delivering at least one infant were eligible for inclusion. Mothers with diseases affecting nutrient metabolism or birthing newborn wards of the state were excluded. Subjects were recruited at delivery for Harvard Food Frequency Questionnaire and plasma sample collection. Plasma carotenoids were analyzed by HPLC-MS. **Results**: A total of 488 patients, including 270 normotensive (NT), 61 CH, 102 GH, and 55 PE, were recruited. Plasma carotenoid analyses were available for 225 subjects. Plasma total, cis-, and trans-β-carotene were significantly lower in PE (73 mcg/L) compared to NT (170 mcg/L), CH (194 mcg/L), and GH (190 mcg/L) groups. Lutein + zeaxanthin and β-cryptoxanthin were also reduced in PE (142 mcg/L and 81 mcg/L) compared to NT (209 mcg/L and 123 mcg/L) but only β-cryptoxanthin was lower in PE compared to GH (126 mcg/L). Levels of α-carotene were lower in PE (18 mcg/L) compared to both CH (43 mcg/L) and GH (48 mcg/L). **Conclusions**: These results demonstrate that plasma carotenoid levels differ among HDP and may suggest that oxidative stress in PE depletes circulating carotenoids, differentiating it from other HDP.

## 1. Introduction

Hypertensive disorders of pregnancy (HDP), including chronic hypertension (CH) during pregnancy, gestational hypertension (GH), preeclampsia (PE), eclampsia, and hemolysis, elevated liver enzymes, low platelet count (HELLP) syndrome, impact one in six pregnancies [1]. HDP are major contributors to maternal and infant morbidity and mortality, accounting for up to 40,000 maternal deaths and 500,000 fetal and newborn deaths annually [2]. Despite their impact, clinical management of HDP remains limited, with delivery as the only definitive treatment [3].

The etiology of HDP is not fully understood; however, oxidative stress is a well-recognized contributor to hypertension and its systemic complications in both pregnant and non-pregnant individuals [4,5]. Increased oxidative stress promotes endothelial dysfunction, an early indicator of impaired vasoreactivity and contributor to HDP pathophysiology [4]. Although a pro-oxidant environment is necessary for normal placental development and parturition, the sustained systemic vasoconstriction observed in HDP from excessive oxidative stress contributes to placental hypoperfusion and altered vascular development [6,7,8]. In HDP, arterial remodeling of the decidual vessels can lead to high-pressure, pulsatile blood flow to the placenta and fetus, exacerbating hypertension and increasing the risk of severe conditions such as PE, eclampsia, and HELLP syndrome [9].

**Figure 1:**
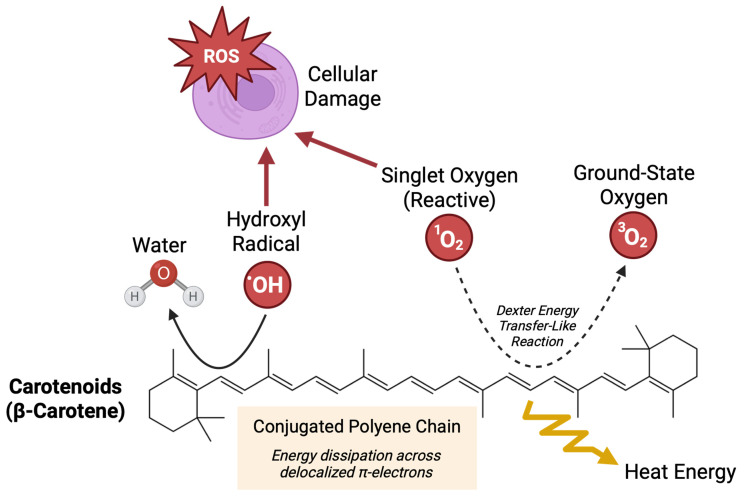
Antioxidative mechanisms of carotenoids. Presence of a polyene chain is a central feature of carotenoids that confers their biological antioxidative activity. This highly conjugated system allows carotenoids to effectively quench reactive oxygen species, including singlet oxygen and free radicals, which can otherwise damage cellular structures. Non-covalent interactions between the π-electron system of the polyene chain and reactive oxygen species facilitate energy dissipation through delocalization and resonance.

Antioxidants are mitigators of oxidative stress. While the human body can produce endogenous antioxidants in response to oxidative stress, dietary intake serves as a crucial source of exogenous antioxidants [10]. Antioxidative nutrients, including carotenoids, have demonstrated therapeutic benefits in conditions characterized by inflammation and oxidative stress [11]. Carotenoids are pigmented tetraterpene derivatives found in plants and algae, but importantly, are not synthesized by humans [12]. Although over 750 carotenoids have been identified in nature, only about 40 are commonly consumed in the human diet, with α-carotene, β-carotene, lycopene, lutein, and β-cryptoxanthin accounting for nearly 90% of dietary intake [13,14]. Carotenoids play diverse biological roles, including modulating inflammation and protecting cells from oxidative stress by neutralizing free radicals and singlet oxygen (Figure 1) [12].

Carotenoid status in humans has been associated with lower blood pressure, and studies have reported reduced carotenoid levels in both plasma and placentae from women with PE [15,16,17,18]. However, there remains a paucity of research evaluating carotenoids in other HDP, including CH and GH. This is a critical gap in knowledge considering the rising rates of both CH and GH and the distinct features of these diagnoses compared to PE [1]. To address this gap, we conducted a retrospective cohort analysis evaluating self-reported maternal dietary carotenoid intake and quantified plasma carotenoid levels in normotensive (NT), CH, GH, and PE pregnancies.

## 2. Materials and Methods

### 2.1. Participant Enrollment

Ethical approval for this prospective cohort study was obtained from the University of Nebraska Medical Center Institutional Review Board (IRB #112-15-EP). Inclusion criteria included women ≥ 19 years old admitted to the Labor and Delivery Unit at Nebraska Medicine (Omaha, NE, USA) for delivery of at least one live-born infant. Exclusion criteria included infants who were legally designated as wards of the state (e.g., placed into foster care) and mothers with diseases affecting normal nutrient metabolism, including gastrointestinal, liver, and kidney diseases and genetic metabolic disorders. The electronic health records of patients admitted to the Labor and Delivery Unit were screened for study recruitment based on these criteria. Study recruitment was coordinated with Labor and Delivery Unit clinicians to ensure patient safety and comfort. All patients provided informed, written consent prior to enrollment in this study at the time of delivery. Study enrollment occurred between the years of 2015 and 2023.

### 2.2. Dietary Questionnaires

Trained study personnel administered demographic questionnaires and the Harvard Food Frequency Questionnaire (FFQ) to maternal participants after admission for labor and delivery [19]. The FFQ queried dietary intake over a three-month period, capturing maternal carotenoid intake during the last trimester of pregnancy. De-identified FFQs were analyzed by the Harvard T.H. Chan School of Public Health to estimate daily intake of carotenoids from foods and supplements. Additional information about the FFQ questionnaire, including quantified nutrients, are included in the data supplement.

### 2.3. Clinical Data Collection

Clinical data was obtained from the maternal electronic medical record. Subject CH status was determined using 2017 ACC/AHA guidelines [20]. Subjects were classified as GH if they had a formal diagnosis in the electronic health record or blood pressure measurements meeting 2017 ACC/AHA guidelines for hypertension recorded after 20 weeks of pregnancy. Subjects were classified as having PE if a formal diagnosis was noted in the electronic medical record. Diagnostic criteria for PE included new-onset hypertension after 20 weeks of pregnancy (systolic blood pressure ≥ 140 mmHg or diastolic blood pressure ≥ 90 mmHg on two occasions at least four hours apart, or systolic blood pressure ≥ 160 mmHg or diastolic blood pressure ≥ 110 mmHg on one occasion) accompanied by either proteinuria (≥300 mg in a 24 h urine collection, protein-to-creatinine ratio ≥ 0.3 mg/dL, or dipstick reading of 2+) or signs of end-organ damage (thrombocytopenia, renal insufficiency, liver dysfunction, pulmonary edema, or refractory new-onset headache) [3]. Subjects were classified as NT if they met none of the above criteria. The final HDP classifications included NT, CH, GH, or PE.

### 2.4. Blood Sample Collection

Maternal blood was collected in K2 EDTA tubes during routine clinical care after admission for labor and delivery. The research team received blood samples leftover from clinical blood draws. Whole blood samples were protected from heat and light in amber-colored zip-top bags stored at 4 °C, separated into plasma and red blood cell components by centrifugation, and frozen at −80 °C within 12 h of collection per the World Health Organization guidelines to preserve nutrient integrity [21].

### 2.5. Carotenoid Analysis

Plasma samples were analyzed for carotenoids (α-carotene, β-carotene, β-cryptoxanthin, combined lutein + zeaxanthin, and lycopene). The Biomarker Research Institute at the Harvard T.H. Chan School of Public Health analyzed samples using high-performance liquid chromatography–mass spectrometry (HPLC-MS) as described by Thoene et al. [21]. All samples were quantified in duplicate. The minimum detection limits of plasma carotenoids were 3.86 μg/L for lutein + zeaxanthin, 3.88 μg/L for β-cryptoxanthin, 5.44 μg/L for lycopene, 4.24 μg/L for α-carotene, and 4.80 μg/L for β-carotene [22]. Quality control was assessed externally by participating in the National Institute of Standards and Technology (NIST) standardization program for carotenoid analysis [23].

### 2.6. Statistical Analysis

Subjects missing HDP or nutrient intake data were excluded from analyses, since these data are key to the central objective of this study. Descriptive statistics, including medians and inner quartiles for continuous variables and frequencies and percentages for categorical variables, were calculated. The normality of continuous variables was assessed by skewness and kurtosis tests. Statistical differences between continuous maternal demographics data were assessed using Kruskal–Wallis tests. Statistical differences between categorical maternal demographics variables were assessed using chi-squared tests. Statistical differences between raw dietary intake and blood levels of carotenoids were assessed using Kruskal–Wallis tests with Dunn’s post-hoc multiple comparisons tests. To account for potential confounding, dietary intake and blood carotenoid levels were log-transformed and examined in exploratory multinomial regression models adjusted for maternal pre-pregnancy BMI and parity. These models are considered exploratory given the limited sample size in blood carotenoid analyses, which may inflate standard errors and reduce the precision of estimates. Summary statistics of demographics data and exploratory multinomial regression analyses were performed using Stata 18.5. Statistical analyses and visualization of raw dietary intake and blood levels of carotenoids was performed using GraphPad Prism (version 10.6.0). The threshold of significance was set at *p*-value < 0.05.

## 3. Results

### 3.1. Maternal Demographics

**Figure 2:**
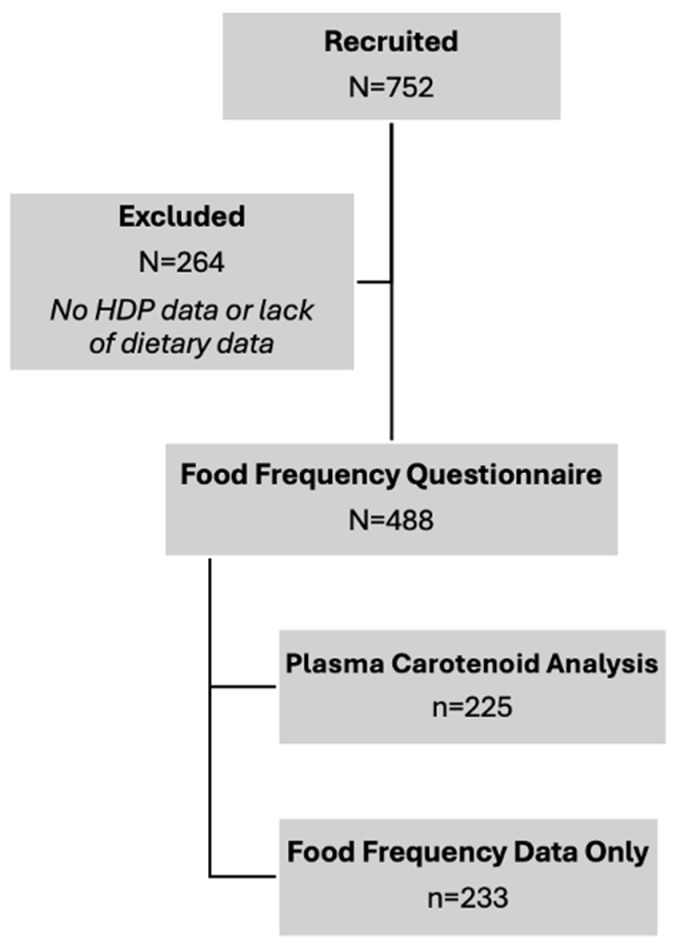
Study flowchart for subject recruitment and analyses. Final sample sizes for carotenoid dietary intake and blood levels are contained.

A total of 488 maternal participants were included in this analysis (Figure 2), of which 270 (55%) were NT, 61 (13%) had CH, 102 (21%) had GH, and 55 (11%) had PE (Table 1). The demographic characteristics of participants were broadly representative of the patient population served at the study site. The age of participants was similar between groups, ranging from 28.6 to 29.6 years. Pre-pregnancy BMI was significantly different between groups, with the lowest BMI medians in the NT and CH groups at 28.3 kg/m^2^ and the highest in the PE group at 33.1 kg/m^2^. BMIs for all groups fell within the overweight (25–29.9) and obese (>30) ranges, consistent with published population demographics for Nebraska women age 20–39 years [24]. The majority of participants identified as white and there were no significant differences in racial composition between HDP groups. Most participants were multiparous at the time of recruitment, although there was a statistically significant difference in parity noted across HDP groups, potentially owing to the higher percentage of nulliparous women in the PE group. There were no significant differences in maternal diabetes status or smoking status between HDP groups. Pregnancy duration was significantly different between HDP groups, with the shortest period in the PE group at 36.4 weeks and the longest in the CH group at 38.9 weeks. Most participants consume carotenoid supplements, as reflected by higher FFQ-derived intake estimates when supplementation was included.

### 3.2. Dietary Carotenoid Intake

**Figure 3:**
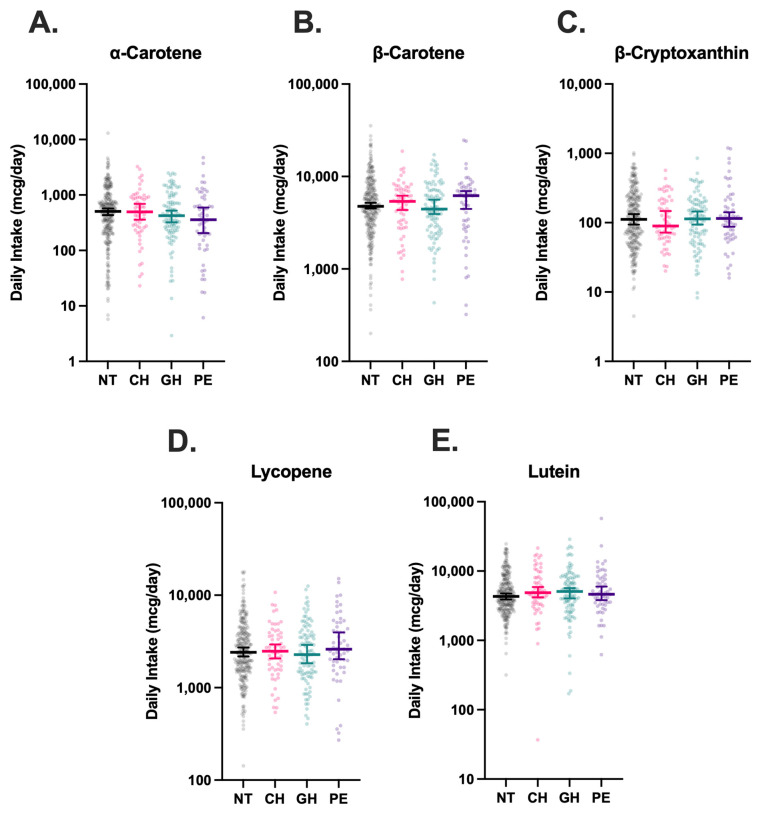
Estimated dietary intake of carotenoids in hypertensive disorders of pregnancy (HDP). Intake of α-carotene (**A**), β-carotene (**B**), β-cryptoxanthin (**C**), Lycopene (**D**), and Lutein (**E**) were stratified by HDP, including normotension (NT, n = 270), chronic hypertension (CH, n = 61), gestational hypertension (GH, n = 102), and preeclampsia (PE, n = 55), and compared using Kruskal–Wallis tests with Dunn’s post-hoc multiple comparisons tests. Vertical brackets indicate 95% confidence intervals. A *p* < 0.05 was considered statistically significant.

Dietary intake data was available for all 488 participants. Comparison of estimated carotenoid intake across groups revealed no statistically significant differences between NT, CH, GH, and PE groups (Figure 3). No significant associations between carotenoid intake and maternal CH, GH, and PE were observed in exploratory multinomial regressions (Table 2).

### 3.3. Plasma Carotenoid Concentrations

**Figure 4:**
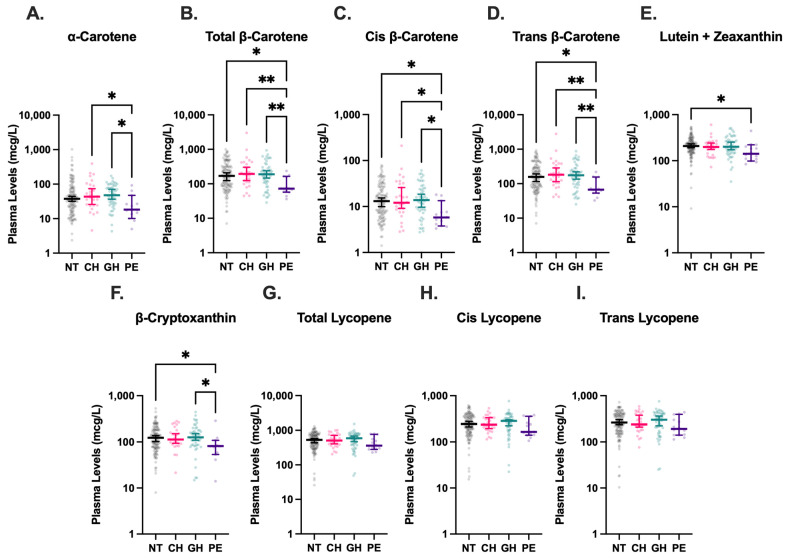
Plasma carotenoid levels in hypertensive disorders of pregnancy (HDP). Blood levels of α-carotene (**A**), total β-carotene (**B**), cis β-carotene (**C**), trans β-carotene (**D**), lutein + zeaxanthin (**E**), β-cryptoxanthin (**F**), total lycopene (**G**), cis lycopene (**H**), and trans lycopene (**I**) were stratified by HDP, including normotension (NT, n = 131), chronic hypertension (CH, n = 28), gestational hypertension (GH, n = 52), and preeclampsia (PE, n = 14), and compared using Kruskal–Wallis tests followed by Dunn’s post hoc multiple comparisons tests. Levels of cis-β-carotene were undetectable in three analyzed plasma samples. Vertical brackets indicate 95% confidence intervals. A *p* < 0.05 was considered statistically significant. * *p* < 0.05, ** *p* < 0.01.

Maternal plasma carotenoid levels were available for 225 of the 488 participants, as detailed in Figure 2, although levels of cis-β-carotene were undetectable in three analyzed plasma samples. Compared to all other HDP groups, women with PE had significantly lower plasma levels of total-β-carotene, cis-β-carotene, and trans-β-carotene (Figure 4). The median concentration of total-β-carotene in the PE group was less than half that observed in the NT group (72.5 mcg/L vs. 169.9 mcg/L, *p* = 0.01). Similarly, the median concentration of cis-β-carotene in PE was less than half of that in NT (5.8 mcg/L vs. 12.9 mcg/L, *p* = 0.01), and trans-β-carotene levels in PE were also markedly lower than in NT (67.1 mcg/L vs. 157.6 mcg/L, *p* = 0.01). Levels of total-β-carotene, cis-β-carotene, and trans-β-carotene were similar among NT, CH, and GH groups, with levels in CH and GH groups also significantly higher than those in PE. Combined lutein + zeaxanthin and β-cryptoxanthin levels were also lower in the PE group compared to the NT group (208.6 mcg/L vs. 141.6 mcg/L, *p* = 0.049 and 123.5 mcg/L vs. 81.1 mcg/L, *p* = 0.048, respectively), although the differences were not as drastic. Levels of β-cryptoxanthin were also significantly lower in PE compared to GH (*p* = 0.02). No significant differences were detected among levels of total, cis-, or trans-lycopene in HDP groups. Significant associations between maternal PE and plasma trans-β-carotene (*p* < 0.001), cis-β-carotene (*p* = 0.047), and total β-carotene (*p* = 0.001) persisted in exploratory multinomial regression analyses (Table 3).

## 4. Discussion

To our knowledge, this is the first study to specifically assess plasma carotenoid status across HDP subtypes. We report significantly decreased levels of previously uncharacterized cis and trans β-carotene isoforms in maternal plasma from pregnancies complicated by PE compared to NT pregnancies. While previous studies have reported lower total β-carotene levels in PE, our results uniquely highlight reductions of both cis and trans β-carotene isoforms, which adds additional resolution in the examination of β-carotene’s role in HDP [17,18,25]. Trans β-carotene is the best absorbed isoform and can undergo isomerization to form cis β-carotene, which has more potent antioxidative properties [26,27]. However, our results showing consistent depletion suggests a generalized perturbation of β-carotene status rather than an isoform-specific effect. Our results also corroborate the findings of previous studies characterizing decreased plasma levels of lutein and β-cryptoxanthin in PE compared to NT pregnancies [16]. Although, interestingly, we did not observe statistically significant differences between α-carotene or lycopene levels in PE and NT, which have been demonstrated in other studies [18,28].

Our study was unique in its inclusion of other HDP, such as CH and GH, in addition to PE, which are relatively understudied in comparison and frequently omitted from investigations of nutrient status during pregnancy. These subtypes are particularly important to study given the rising rates of HDP risk factors, GH, and CH among women of childbearing age [1]. Despite this broader inclusion, we did not observe significant associations between carotenoid levels and these other HDP subtypes. This distinction may point toward fundamental differences in redox state between PE and other HDP conditions. The increased oxidative stress characteristic of PE may lead to greater consumption of carotenoids in the process of quenching ROS, resulting in lower circulating levels [4,29]. In contrast, the oxidative stress present in CH or GH may not be as severe, and thus, may not exert a measurable impact on plasma carotenoid levels. This hypothesis is supported by the significantly lower plasma α-carotene, β-carotene, and β-cryptoxanthin in PE compared to GH and CH, which both fall on the HDP spectrum, but have notably milder clinical features than PE.

Clinical trials evaluating targeted carotenoid supplementation, specifically lycopene supplementation, have not demonstrated meaningful benefit in preventing PE [30]. However, this is important to consider in the context that our data show no significant differences in plasma lycopene levels between NT and PE groups. It is possible that levels of plasma lycopene are different between NT and PE groups early in pregnancy as opposed to the end of pregnancy when we conducted our analysis. It is also possible that other carotenoids, or more likely, a synergism of multiple carotenoids could have meaningful clinical impacts in reducing PE occurrence if supplemented during early pregnancy. However, the relationship between carotenoid status and PE risk may be more complex than intake alone. Most participants (>83% across all HDP groups) had some degree of carotenoid supplementation, explainable by the frequent inclusion of pro-vitamin A carotenoids in prenatal and daily multivitamins. Despite lower levels of plasma carotenoids in our PE group, our dietary intake data did not reveal reduced carotenoid intake among individuals with PE compared to any other HDP group, which was true of both total intake and intake without supplements (Figure S1). This differs from previous studies, such as Kang et al., who reported an association between decreased dietary intake of β-carotene and lutein + zeaxanthin and the development of PE [31]. Notably, we found no meaningful correlations between maternal dietary carotenoid intake and measured plasma levels except for α-carotene (Figure S2). While carotenoid intake data was estimated from self-reported dietary questionnaires, this observation suggests that reduced intake may not fully explain the lower carotenoid levels observed in PE. Several factors are known to influence carotenoid bioavailability, including lipid absorption, gut microbiota composition, genetic polymorphisms, and dietary fat co-ingestion, which may also contribute to PE [13,32,33,34]. Future studies may explore the coordination of these factors to better understand the complex milieu influencing carotenoid bioavailability and HDP development.

Other studies have evaluated maternal plasma carotenoid levels during PE as biomarkers for the redox environment, with evidence suggesting that mid-pregnancy α-carotene, β-carotene, and lutein plasma levels may be suitable targets [28]. Our data also support the utility of carotenoids as markers of PE, but with notable caveats, including assessment at delivery and incompletely characterized trends in plasma carotenoid levels over the course of pregnancy. There are known positive correlations between gestational age and maternal plasma carotenoid concentrations, which were also observed in our dataset (Figure S3) [23]. However, it remains unclear whether this relationship is prognostic for earlier delivery or variation in carotenoid metabolism over gestation [23]. To clarify this, future studies should consider serial measurement of maternal carotenoids throughout pregnancy to better define their temporal dynamics and potential utility as early indicators for the risk of developing HDP.

The prevalence of HDP in our study was notably higher than regional estimates, at 45% compared to 15% across the Midwestern United States [35]. The reasons for this discrepancy may be due to the extended period of admission to the Labor and Delivery Unit, frequent in complicated pregnancies. This additional time in the unit provides greater opportunity to recruit patients, administer demographics surveys and FFQs, and collect plasma samples. Moreover, UNMC is a major regional medical center with a level III Neonatal Intensive Care Unit and a frequent referral center for complicated pregnancies. These factors may have artificially inflated the prevalence of HDP in our cohort and skewed our results towards complicated pregnancies. However, the numerous NT mothers (n = 270) and uncomplicated pregnancies included in our cohort support the validity of our results with respect to comparative carotenoid levels among HDP. Additional data collected using unbiased recruitment strategies is necessary to determine a precise estimate of HDP prevalence in Eastern Nebraska.

Our study was limited by sample size, which reduced statistical power and precluded the use of robust multivariate regression models as a primary analysis method. We acknowledge that our PE sample size, in particular, was limited, with as few as fourteen participants in plasma carotenoid analyses. Adjusted regression models are sensitive to overfitting when the number of covariates exceeds the degrees of freedom allowed by the sample size, as in our dataset [36]. Adjusted analyses can strengthen our interpretations, as factors such as obesity, parity, and maternal age are known to influence HDP risk [37,38]. Therefore, we conducted exploratory regression analyses, adjusted for maternal BMI and parity, which corroborated the presented carotenoid intake and plasma β-carotene results, although these must be interpreted cautiously. Additionally, the study design involving at-delivery recruitment and data collection restricts causal inferences regarding the impacts of carotenoids on HDP development. Rather, our results capture a snapshot of carotenoid status during the peripartum period, when maternal and newborn outcomes are most immediately affected. Despite these limitations, our study had notable strengths, including the assessment of both dietary intake and plasma carotenoid levels, characterization of a broad spectrum of carotenoids, and the inclusion of under-studied HDP subtypes such as GH and CH during pregnancy. These findings contribute to the growing body of evidence implicating the role of carotenoids in the pathophysiology of PE and provide important distinctions between specific HDP categories. Future studies should evaluate the synergistic impact of carotenoids in ameliorating oxidative states and the temporal dynamics of plasma carotenoid levels during pregnancy to assess the utility of carotenoids as therapeutic targets and biomarkers for PE.

## 5. Conclusions

Maternal plasma carotenoids, including β-carotene, lutein + zeaxanthin, and β-cryptoxanthin, were lower in PE compared to NT. Plasma α-carotene, β-carotene, and β-cryptoxanthin were also lower in PE compared to GH, and plasma α-carotene and β-carotene were lower in PE compared to CH. These data may indicate that PE is a unique redox state compared to other HDP, potentially requiring greater quantities of carotenoids to quench ROS. Despite differences in plasma carotenoid levels, dietary intake of carotenoids did not differ between HDP groups, suggesting that intake is not the sole determinant of carotenoid bioavailability. These observations highlight the role of carotenoids in PE, specifically. Clarifying whether and how carotenoid status influences PE risk will require mechanistic and longitudinal studies across pregnancy.

## Figures and Tables

**Table 1:** Demographics information.

	NT (n = 270)	CH (n = 61)	GH (n = 102)	PE (n = 55)	*p*-Value
Age (years; median, inner quartiles)	30 (25–33)	30 (25–33)	30 (25–34)	28 (23–34)	0.7000
Body Mass Index (kg/m^2^; median, inner quartiles)	27.3 (23.4–31.8)	26.8 (22.4–33.2)	28.0 (22.9–32.4)	34.2 (25.7–38.8)	0.0007
Race (N, %)					0.295
White	180 (66.9%)	46 (75.4%)	73 (71.6%)	37 (67.3%)	
African American	40 (14.9%)	6 (9.8%)	9 (8.8%)	8 (14.6%)	
Hispanic	22 (8.2%)	2 (3.3%)	7 (6.7%)	3 (5.5%)	
Asian or Pacific Islander	6 (2.2%)	3 (4.9%)	1 (1.0%)	2 (3.6%)	
American Indian	-	1 (1.6%)	-	-	
Other/Unknown	21 (7.8%)	3 (4.9%)	12 (11.8%)	5 (9.1%)	
Parity (N, %)					0.024
Nulliparous	31 (11.5%)	4 (6.6%)	11 (10.8%)	14 (25.5%)	
Primiparous	83 (30.7%)	24 (39.3%)	37 (36.3%)	20 (36.4%)	
Multiparous	156 (57.8%)	33 (54.1%)	54 (52.9%)	21 (38.2%)	
Diabetes (N, %)	34 (12.6%)	4 (6.6%)	10 (9.8%)	11 (20.0%)	0.135
Smoking (N, %)					0.297
Current	28 (10.4%)	5 (8.2%)	7 (6.9%)	5 (9.1%)	
Former	32 (11.9%)	5 (8.2%)	17 (16.7%)	12 (21.2%)	
Pregnancy Duration (weeks; median, inner quartiles)	39.3 (38.4–40.2)	39.4 (38.3–40.4)	39.2 (38–40.3)	37 (34.1–38.6)	0.0001
Carotenoid Supplementation (N, %)	93.0% (251)	85.3% (52)	90.2% (92)	83.6% (46)	0.079

Subjects were stratified by the hypertensive disorders of pregnancy (HDP), including normotension (NT), chronic hypertension (CH), gestational hypertension (GH), preeclampsia (PE). Statistical comparisons between HDP group continuous demographics variables were performed using Kruskal–Wallis tests. Statistical comparisons between HDP group categorical variables were performed using chi-squared tests. A *p* < 0.05 was considered statistically significant.

**Table 2:** Exploratory multinomial regression of estimated carotenoid intake and hypertensive disorders of pregnancy (HDP).

	CH (n = 61)	GH (n = 95)	PE (n = 49)
α-carotene	1.03 (0.81–1.32)	0.94 (0.77–1.14)	0.86 (0.66–1.13)
β-carotene	0.96 (0.65–1.42)	0.85 (0.62–1.17)	0.78 (0.50–1.23)
β-cryptoxanthin	0.91 (0.67–1.23)	0.88 (0.68–1.16)	0.99 (0.69–1.41)
Lycopene	1.14 (0.71–1.83)	1.02 (0.72–1.45)	1.09 (0.74–1.62)
Lutein	0.95 (0.67–1.36)	0.84 (0.60–1.17)	1.03 (0.64–1.67)

Associations between log-transformed carotenoid intake and risk of HDP, including normotension (NT, n = 270), chronic hypertension (CH, n = 61), gestational hypertension (GH, n = 102), preeclampsia (PE, n = 55). Estimates derived from multinomial logistic regression adjusted for maternal BMI and parity. Results presented as relative risk ratios (RRRs) per log_10_ unit increase with 95% confidence intervals.

**Table 3:** Exploratory multinomial regression of plasma carotenoid levels and hypertensive disorders of pregnancy (HDP).

	CH (n = 28)	GH (n = 49)	PE (n = 11)
**Lutein + Zeaxanthin**	0.68 (0.29–1.62)	1.31 (0.60–2.86)	0.67 (0.18–2.46)
**β-cryptoxanthin**	0.94 (0.49–1.81)	1.17 (0.63–2.16)	0.63 (0.27–1.48)
**Trans Lycopene**	1.07 (0.57–2.00)	1.34 (0.71–2.52)	0.83 (0.34–2.01)
**Cis Lycopene**	1.19 (0.65–2.17)	1.35 (0.68–2.67)	1.06 (0.41–2.74)
**Total Lycopene**	1.13 (0.61–2.12)	1.35 (0.69–2.65)	0.91 (0.36–2.32)
**α-carotene**	1.14 (0.74–1.77)	1.30 (0.94–1.81)	0.65 (0.33–1.26)
**Trans β-carotene**	1.22 (0.75–1.98)	1.22 (0.84–1.76)	0.35 (0.19–0.62) ***
**Cis β-carotene**	1.10 (0.63–1.91)	1.20 (0.82–1.76)	0.42 (0.18–0.99) *
**Total β-carotene**	1.21 (0.74–1.99)	1.23 (0.85–1.78)	0.35 (0.19–0.64) **

Associations between log-transformed plasma carotenoid levels and risk of HDP, including normotension (NT, n = 270), chronic hypertension (CH, n = 61), gestational hypertension (GH, n = 102), preeclampsia (PE, n = 55). Estimates derived from multinomial logistic regression adjusted for maternal BMI and parity. Results presented as relative risk ratios (RRRs) per log_10_ unit increase with 95% confidence intervals. * *p* < 0.05, ** *p* < 0.01, *** *p* < 0.001.

## Data Availability

De-identified data and analysis files are provided in the Supplementary Materials. The authors can provide data in alternative formats upon request.

## Supplementary Materials

The following supporting information can be downloaded at: https://www.mdpi.com/article/10.3390/nu17193104/s1, Figure S1: Dietary intake of carotenoids without supplementation in HDP; Figure S2: Maternal plasma carotenoid levels compared to dietary carotenoid intake; Figure S3: Maternal plasma carotenoid levels compared to gestational age; analysis files and raw Data.

## Abbreviations

The following abbreviations are used in this manuscript:

HDP	Hypertensive disorders of pregnancy
ROS	Reactive oxygen species
GH	Gestational hypertension
CH	Chronic hypertension
PE	Preeclampsia
NT	Normotension
HELLP	Hemolysis, elevated liver enzymes, low platelet count
FFQ	Food frequency questionnaire
