# Supplementary material for: Differential Plasma Carotenoid Profiles in Hypertensive Disorders of Pregnancy
Source: Nutrients. 2025 Sep 29;17(19):3104. doi: 10.3390/nu17193104 (PMC12525936; doi:10.3390/nu17193104)
Supplement: Supplementary file 1 [file nutrients-17-03104-s001.zip › Supplementary Information.pdf]

Supplementary Materials

Additional information regarding the Harvard Food Frequency Questionnaires (FFQ) can be found online at https://hsph.harvard.edu/department/nutrition/nutrition-questionnaire-service-center/. The semi-quantitative FFQ has been developed over 40 years and validated for use in adult populations. This survey instrument assesses dietary nutrient intake over a three-month period. Blank questionnaire bubble sheets are available through the Harvard T.H. Chan School of Public Health Department of Nutrition. Questionnaires take approximately twenty minutes to complete. All questionnaire forms are mailed for analysis by the Harvard T.H. Chan School of Public Health Department of Nutrition. Results, including estimates of 227 nutrient levels, are delivered in a Microsoft Excel spreadsheet.

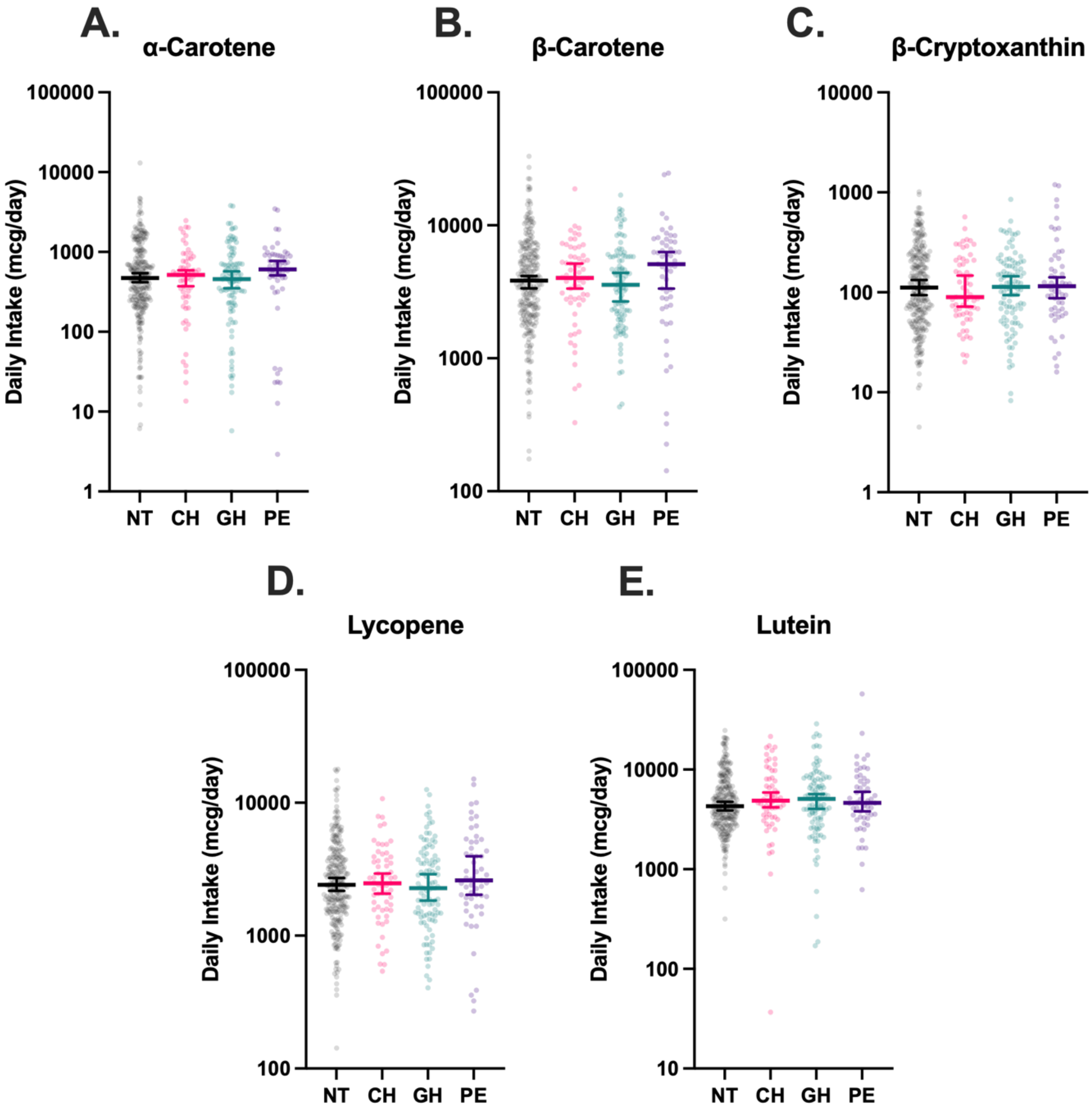

Figure S1. Estimated Dietary Intake of Carotenoids Without Supplementation in Hypertensive Disorders of Pregnancy (HDP). Intake of α-carotene (A), β-carotene (B), β-cryptoxanthin (C), Lycopene (D), and Lutein (E) were stratified by HDP, including normotension (NT, n = 270), chronic hypertension (CH, n = 61), gestational hypertension (GH, n = 102), and preeclampsia (PE, n = 55), and compared using Kruskal–Wallis tests with Dunn's post-hoc multiple comparisons tests. A $p<0.05$ was considered statistically significant.

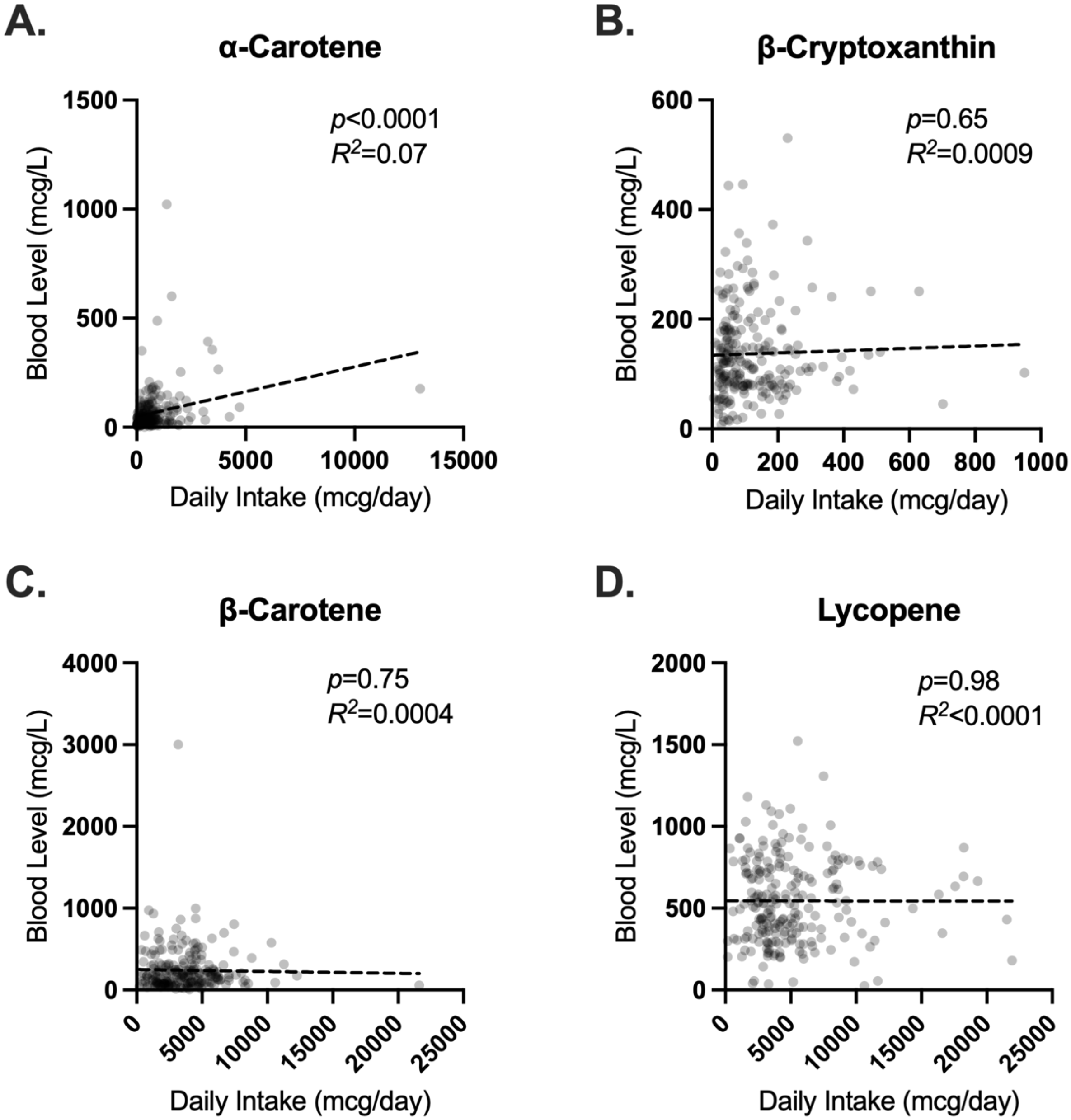

Figure S2. Maternal Plasma Carotenoid Levels Compared to Dietary Carotenoid Intake. Maternal plasma carotenoid concentrations were measured in samples collected at delivery using high-performance liquid chromatography coupled with mass spectrometry (HPLC-MS). Maternal dietary intake of carotenoids was assessed using Food Frequency Questionnaires at the time of delivery. Plasma levels of α-carotene (A), β-carotene (B), β-cryptoxanthin (C), Lycopene were plotted against dietary intake and Spearman's Correlation was conducted to assess significance with a threshold of $p<0.05$.

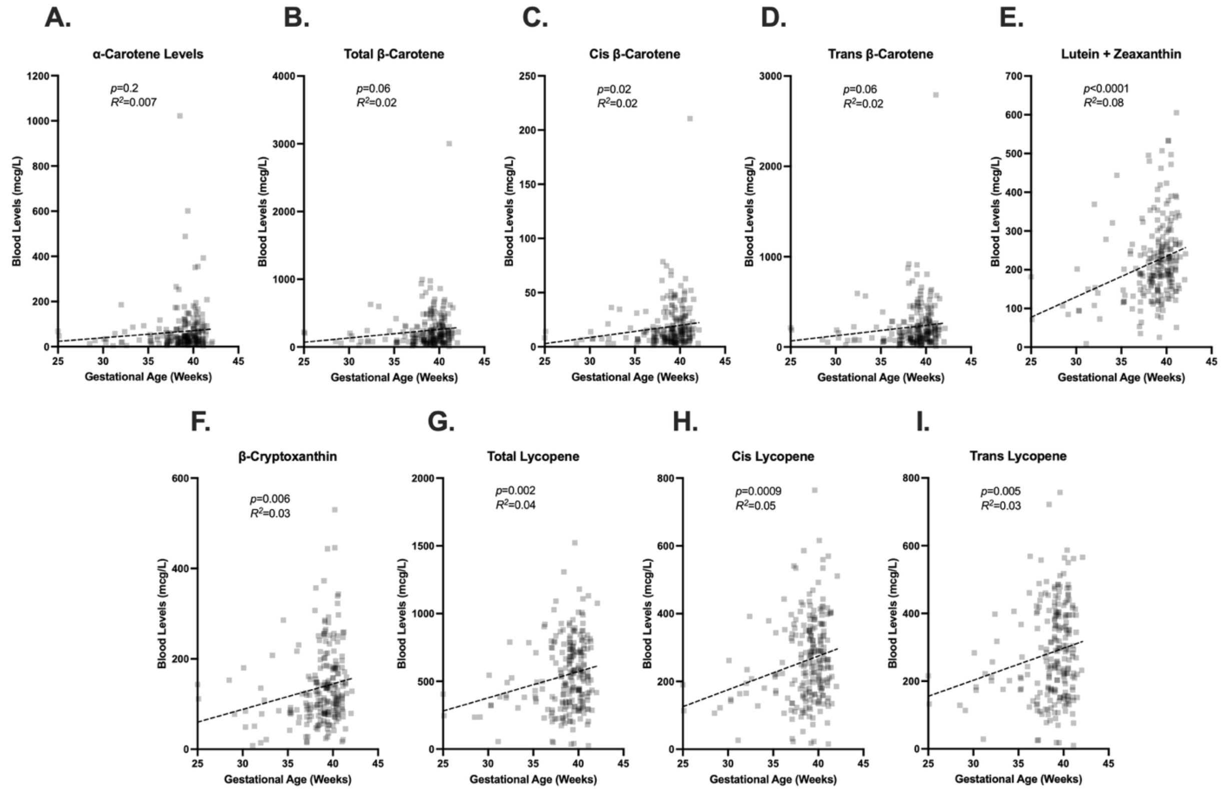

Figure S3. Maternal Plasma Carotenoid Levels Compared to Gestational Age. Maternal plasma carotenoid concentrations were measured in samples collected at delivery using high-performance liquid chromatography coupled with mass spectrometry (HPLC-MS). Gestational age for each participant was retrieved from the electronic health record. Plasma levels of α-carotene (A), total β-carotene (B), cis β-carotene (C), trans β-carotene (D), lutein + zeaxanthin (E), β-cryptoxanthin (F), total lycopene (G), cis lycopene (H), and trans lycopene (I) were plotted against gestational age and Spearman's Correlation was conducted to assess significance with a threshold of $p<0.05$.
