# Supplementary material for: Differential Plasma Carotenoid Profiles in Hypertensive Disorders of Pregnancy
Source: Nutrients. 2025 Sep 29;17(19):3104. doi: 10.3390/nu17193104 (PMC12525936; doi:10.3390/nu17193104)
Supplement: Supplementary file 1 [file nutrients-17-03104-s001.zip › Supplementary Figures/Figure S3.pdf]

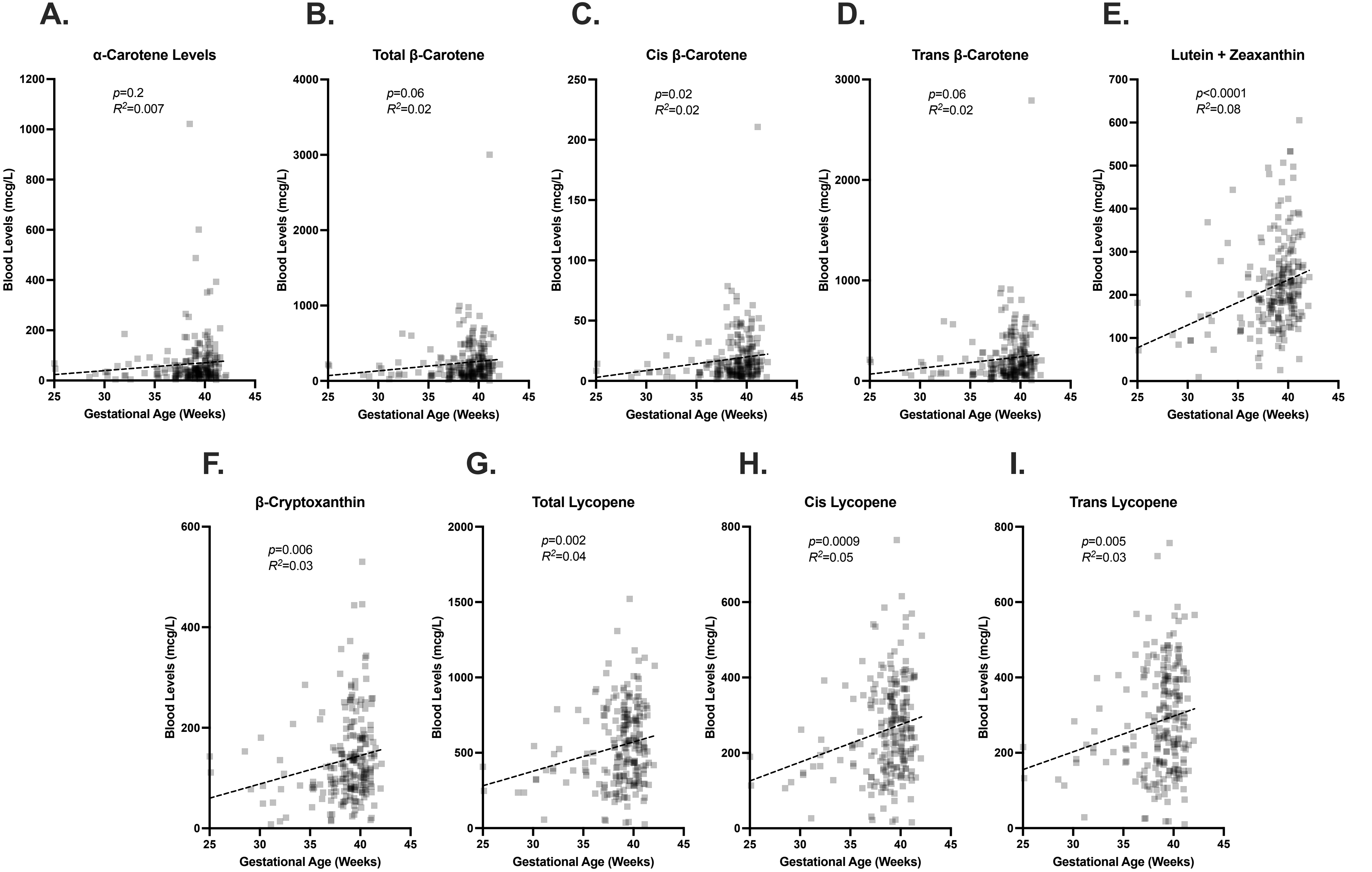

A.
α-Carotene Levels
p=0.2
R²=0.007
B.
Total β-Carotene
p=0.06
R²=0.02
C.
Cis β-Carotene
p=0.02
R²=0.02
D.
Trans β-Carotene
p=0.06
R²=0.02
E.
Lutein + Zeaxanthin
p<0.0001
R²=0.08
F.
β-Cryptoxanthin
p=0.006
R²=0.03
G.
Total Lycopene
p=0.002
R²=0.04
H.
Cis Lycopene
p=0.0009
R²=0.05
I.
Trans Lycopene
p=0.005
R²=0.03
Blood Levels (mcg/L)
Gestational Age (Weeks)
