# Supplementary material for: Differential Plasma Carotenoid Profiles in Hypertensive Disorders of Pregnancy
Source: Nutrients. 2025 Sep 29;17(19):3104. doi: 10.3390/nu17193104 (PMC12525936; doi:10.3390/nu17193104)
Supplement: Supplementary file 1 [file nutrients-17-03104-s001.zip › Carotenoids in HDP.pdf]

```
      name:  <unnamed>
       log:  /Users/colmanfreel/Downloads/Carotenoids in HDP.smcl
  log type:  smcl
 opened on:   8 Sep 2025, 22:48:42

 1 . do "/var/folders/st/l4cwsv_d4452ngyk_vnsb0rr0000gn/T//SD36082.000000"

 2 . *prepared by Colman Freel (cfreel@unmc.edu) on March 27th, 2025
 3 . *upated by Colman Freel (cfreel)@unmc.edu on May 30th, 2025
 4 . *updated by Colman Freel (cfreel@unmc.edu) on July 23rd, 2025
 5 . *updated by Colman Freel (cfreel@unmc.edu) on August 4th, 2025
 6 . *updated by Colman Freel (cfreel@unmc.edu) on September 8th, 2025
 7 .
 8 . *importing data (change to your file path)
 9 .
10 . import excel "/Users/colmanfreel/Documents/Research/Graduate Research/Carot
   > enoids in HDP/Carotenoids in HDP.xlsx", sheet("Carotenoid Master List") fir
   > strow case(lower)
   (28 vars, 488 obs)

11 .
12 . *examining variables
13 .
14 . destring ga, replace
   ga: all characters numeric; replaced as double
   (7 missing values generated)

15 . sum htncategoryaccaha
```

Variable	Obs	Mean	Std. dev.	Min	Max
htncategor~a	**488**	**1.881148**	**1.096107**	**1**	**4**

16 . lab define htncategoryaccaha 1 "NT" 2 "CH" 3 "GH" 4 "PE"

17 . lab val htncategoryaccaha htncategoryaccaha

18 . lab var htncategoryaccaha "HDP Status"

19 . sum age

Variable	Obs	Mean	Std. dev.	Min	Max
age	**486**	**29.40947**	**5.440497**	**19**	**44**

20 . replace age=. if age==0
(0 real changes made)

21 . lab var age "Maternal Age in Years"

22 . sum bmi

Variable	Obs	Mean	Std. dev.	Min	Max
bmi	**460**	**28.91261**	**7.256215**	**16.47**	**60.8**

23 . lab var bmi "Maternal BMI"

24 . replace bmi=. if bmi==0
(0 real changes made)

25 . sum race

Variable	Obs	Mean	Std. dev.	Min	Max
race	**487**	**1.772074**	**1.475242**	**1**	**6**

```
26 . replace race=. if race==0
   (0 real changes made)

27 . lab define race 1 "White" 2 "African American" 3 "Hispanic" 4 ///
   >         "Asian or Pacific Islander" 5 "American Indian" 6 "Other/Unknown"

28 . lab val race race

29 . lab var race "Maternal Race"

30 . sum parity

       Variable |        Obs        Mean    Std. dev.       Min        Max
   -------------+---------------------------------------------------------
         parity |        488    1.862705    1.396535          0          8

31 . lab var parity "Maternal Parity"

32 . sum maternaldiabetes

       Variable |        Obs        Mean    Std. dev.       Min        Max
   -------------+---------------------------------------------------------
   maternaldi~s |        488    .1209016    .3263475          0          1

33 . lab define maternaldiabetes 0 "No Diabetes" 1 "Diabetes"

34 . lab val maternaldiabetes maternaldiabetes

35 . lab var maternaldiabetes "Maternal Diabetes Status"

36 . sum smokingstatus

       Variable |        Obs        Mean    Std. dev.       Min        Max
   -------------+---------------------------------------------------------
   smokingsta~s |        488    .3627049    .7089936          0          2
```

```
37 . lab define smokingstatus 0 "Never" 1 "Current" 2 "Former"

38 . lab val smokingstatus smokingstatus

39 . lab var smokingstatus "Maternal Smoking Status"

40 . lab var ga "Gestational Age"

41 . sum ga
```

Variable	Obs	Mean	Std. dev.	Min	Max
ga	**481**	**38.47131**	**2.540564**	**25**	**42.1**

```
42 .
43 . *generating parity bins
44 .
45 . gen par=0

46 . replace par=1 if parity==1
   (164 real changes made)

47 . replace par=2 if parity>1
   (264 real changes made)

48 . replace par=. if parity==.
   (0 real changes made)

49 . lab define par 0 "Nulliparous" 1 "Primiparous" 2 "Multiparous"

50 . lab val par par

51 . lab var par "Maternal Parity Bins"
```

```
52 .
53 . *examining dietary intake variabels
54 .
55 . sum acar

    Variable |        Obs        Mean    Std. dev.       Min        Max
-------------+---------------------------------------------------------
        acar |        488    704.1723    907.0587       2.92   13007.14

56 . sktest acar

   Skewness and kurtosis tests for normality
                                                         ------ Joint test ----
   > --
    Variable |       Obs   Pr(skewness)   Pr(kurtosis)   Adj chi2(2)  Prob>ch
   > i2
-------------+-----------------------------------------------------------------
   > --
        acar |       488         0.0000         0.0000        462.34     0.00
   > 00

57 . hist acar
   (bin=22, start=2.92, width=591.10091)

58 . lab var acar "Maternal total a-carotene intake"

59 .
60 . sum acar_wo

    Variable |        Obs        Mean    Std. dev.       Min        Max
-------------+---------------------------------------------------------
     acar_wo |        488    730.0324    914.4018       2.92   13007.14

61 . sktest acar_wo

   Skewness and kurtosis tests for normality
                                                         ------ Joint test ----
   > --
    Variable |       Obs   Pr(skewness)   Pr(kurtosis)   Adj chi2(2)  Prob>ch
   > i2
-------------+-----------------------------------------------------------------
   > --
     acar_wo |       488         0.0000         0.0000        456.54     0.00
   > 00
```

```
62 . hist acar_wo
   (bin=22, start=2.92, width=591.10091)

63 . lab var acar_wo "Maternal a-carotene intake without supplements"

64 .
65 . sum bcar

    Variable |        Obs        Mean    Std. dev.       Min        Max
-------------+---------------------------------------------------------
        bcar |        488    5846.083    4288.583     200.37   35404.36

66 . sktest bcar

   Skewness and kurtosis tests for normality
                                                        ----- Joint test ---
   > --
    Variable |       Obs   Pr(skewness)   Pr(kurtosis)   Adj chi2(2)  Prob>ch
   > i2
-------------+---------------------------------------------------------------
   > --
        bcar |       488         0.0000         0.0000        193.77     0.00
   > 00

67 . hist bcar
   (bin=22, start=200.37, width=1600.1814)

68 . lab var bcar "Maternal total b-carotene intake"

69 .
70 . sum bcar_wo

    Variable |        Obs        Mean    Std. dev.       Min        Max
-------------+---------------------------------------------------------
     bcar_wo |        488    4908.752    4085.788     142.38   33004.36
```

```
71 . sktest bcar_wo

   Skewness and kurtosis tests for normality
                                                         ───── Joint test ───
   > ──
       Variable │       Obs   Pr(skewness)   Pr(kurtosis)   Adj chi2(2)  Prob>ch
   > i2
   ─────────────┼──────────────────────────────────────────────────────────────
   > ──
        bcar_wo │       488         0.0000         0.0000        212.01     0.00
   > 00

72 . hist bcar_wo
   (bin=22, start=142.38, width=1493.7264)

73 . lab var bcar_wo "Maternal b-carotene intake without supplements"

74 .
75 . sum bcryp

       Variable │        Obs        Mean    Std. dev.       Min        Max
   ─────────────┼─────────────────────────────────────────────────────────
          bcryp │        488    163.8578    164.2085       4.48    1194.32

76 . sktest bcryp

   Skewness and kurtosis tests for normality
                                                         ───── Joint test ───
   > ──
       Variable │       Obs   Pr(skewness)   Pr(kurtosis)   Adj chi2(2)  Prob>ch
   > i2
   ─────────────┼──────────────────────────────────────────────────────────────
   > ──
          bcryp │       488         0.0000         0.0000        215.37     0.00
   > 00
```

```
77 . hist bcryp
   (bin=22, start=4.48, width=54.083636)

78 . lab var bcryp "Maternal total b-cryptoxanthin intake"

79 .
80 . sum bcryp_wo

    Variable |        Obs        Mean    Std. dev.       Min        Max
-------------+---------------------------------------------------------
    bcryp_wo |        488    163.8586    164.2083       4.48    1194.32

81 . sktest bcryp_wo

   Skewness and kurtosis tests for normality
                                                     ----- Joint test ---
   > --
    Variable |       Obs   Pr(skewness)   Pr(kurtosis)   Adj chi2(2)  Prob>ch
   > i2
-------------+-------------------------------------------------------------
   > --
    bcryp_wo |       488         0.0000         0.0000        215.37     0.00
   > 00

82 . hist bcryp_wo
   (bin=22, start=4.48, width=54.083636)

83 . lab var bcryp_wo "Maternal b-cryptoxanthin intake without supplements"

84 .
85 . sum lut

    Variable |        Obs        Mean    Std. dev.       Min        Max
-------------+---------------------------------------------------------
         lut |        488    3191.224    2634.732     142.82   17866.67
```

```
86 . sktest lut

   Skewness and kurtosis tests for normality
                                                          ───── Joint test ───
   > ──
       Variable │       Obs   Pr(skewness)   Pr(kurtosis)   Adj chi2(2)  Prob>ch
   > i2
   ─────────────┼──────────────────────────────────────────────────────────────
   > ──
            lut │       488         0.0000         0.0000        198.80     0.00
   > 00

87 . hist lut
   (bin=22, start=142.82, width=805.62955)

88 . lab var lut "Maternal total lutein intake"

89 .
90 . sum lut_wo

       Variable │        Obs        Mean    Std. dev.       Min        Max
   ─────────────┼─────────────────────────────────────────────────────────
         lut_wo │        488    3190.177     2634.44     142.82   17866.67

91 . sktest lut_wo

   Skewness and kurtosis tests for normality
                                                          ───── Joint test ───
   > ──
       Variable │       Obs   Pr(skewness)   Pr(kurtosis)   Adj chi2(2)  Prob>ch
   > i2
   ─────────────┼──────────────────────────────────────────────────────────────
   > ──
         lut_wo │       488         0.0000         0.0000        198.92     0.00
   > 00
```

```
 92 . hist lut_wo
    (bin=22, start=142.82, width=805.62955)

 93 . lab var lut_wo "Maternal lutein intake without supplements"

 94 .
 95 . sum lyco

    Variable |        Obs        Mean    Std. dev.       Min        Max
-------------+---------------------------------------------------------
        lyco |        487    6022.306     4976.41      36.75   57276.38

 96 . sktest lyco

    Skewness and kurtosis tests for normality
                                                        ------ Joint test ----
> --
    Variable |       Obs   Pr(skewness)   Pr(kurtosis)   Adj chi2(2)  Prob>ch
> i2
-------------+----------------------------------------------------------------
> --
        lyco |       487         0.0000         0.0000        305.43     0.00
> 00

 97 . hist lyco
    (bin=22, start=36.75, width=2601.8014)

 98 . lab var lyco "Maternal total lycopene intake"

 99 .
100 . sum lyco_wo

    Variable |        Obs        Mean    Std. dev.       Min        Max
-------------+---------------------------------------------------------
     lyco_wo |        487    6019.226    4975.688      36.75   57276.38
```

```
101 . sktest lyco_wo

    Skewness and kurtosis tests for normality
                                                          ───── Joint test ───
    > ──
        Variable │       Obs   Pr(skewness)   Pr(kurtosis)   Adj chi2(2)  Prob>ch
    > i2
    ─────────────┼──────────────────────────────────────────────────────────────
    > ──
         lyco_wo │       487         0.0000         0.0000        305.55     0.00
    > 00

102 . hist lyco_wo
    (bin=22, start=36.75, width=2601.8014)

103 . lab var lyco_wo "Maternal lycopene intake without supplements"

104 .
105 . *examining maternal blood level variables
106 .
107 . sum maternalluteinzeaxanthinmc

        Variable │        Obs        Mean    Std. dev.       Min        Max
    ─────────────┼─────────────────────────────────────────────────────────
    maternallu~c │        225    219.9945    100.4032       9.17     605.45

108 . sktest maternalluteinzeaxanthinmc

    Skewness and kurtosis tests for normality
                                                                          ─────
    > Joint test ─────
                      Variable │       Obs   Pr(skewness)   Pr(kurtosis)   Adj ch
    > i2(2)  Prob>chi2
    ───────────────────────────┼────────────────────────────────────────────────
    > ────────────────
    maternalluteinzeaxanthinmc │       225         0.0000         0.0020
    > 30.58
    >           0.0000
```

```
109 . hist maternalluteinzeaxanthinmc
    (bin=15, start=9.17, width=39.752)

110 . lab var maternalluteinzeaxanthinmc "Maternal lutein and zeaxanthin blood le
    > vels"

111 .
112 . sum maternalbcryptoxanthinmcgl

        Variable |        Obs        Mean    Std. dev.       Min        Max
    -------------+---------------------------------------------------------
    maternalbc~l |        225     136.889    83.29638       7.96      530.1

113 . sktest maternalbcryptoxanthinmcgl

    Skewness and kurtosis tests for normality
                                                                         -----
    > Joint test ------
                      Variable |       Obs   Pr(skewness)   Pr(kurtosis)   Adj ch
    > i2(2)  Prob>chi2
    ---------------------------+---------------------------------------------------
    > ----------------
    maternalbcryptoxanthinmcgl |       225         0.0000         0.0000
    > 50.82
    >          0.0000

114 . hist maternalbcryptoxanthinmcgl
    (bin=15, start=7.96, width=34.809333)

115 . lab var maternalbcryptoxanthinmcgl "Maternal b-cryptoxanthin blood levels"

116 .
117 . sum maternaltranslycopenemcgl

        Variable |        Obs        Mean    Std. dev.       Min        Max
    -------------+---------------------------------------------------------
    maternaltr.. |        225    284.2867    137.9816      10.33     757.08
```

```
118 . sktest maternaltranslycopenemcgl

    Skewness and kurtosis tests for normality
                                                                 ───── J
    > oint test ─────
                     Variable │      Obs   Pr(skewness)   Pr(kurtosis)   Adj chi
    > 2(2)  Prob>chi2
    ──────────────────────────┼──────────────────────────────────────────────────
    > ───────────────
    maternaltranslycopenemcgl │      225         0.0113         0.8206
    > 6.26
    >          0.0437

119 . hist maternaltranslycopenemcgl
    (bin=15, start=10.33, width=49.783333)

120 . lab var maternaltranslycopenemcgl "Maternal trans lycopene blood levels"

121 .
122 . sum maternalcislycopenemcgl

        Variable │        Obs        Mean    Std. dev.       Min        Max
    ─────────────┼─────────────────────────────────────────────────────────
    maternalci.. │        225    261.1442    123.3561      15.48     764.62

123 . sktest maternalcislycopenemcgl

    Skewness and kurtosis tests for normality
                                                               ───── Joi
    > nt test ─────
                   Variable │      Obs   Pr(skewness)   Pr(kurtosis)   Adj chi2(
    > 2)  Prob>chi2
    ────────────────────────┼────────────────────────────────────────────────────
    > ─────────────
    maternalcislycopenemcgl │      225         0.0003         0.0524         14.
    > 22
    >        0.0008
```

```
124 . hist maternalcislycopenemcgl
    (bin=15, start=15.48, width=49.942667)

125 . lab var maternalcislycopenemcgl "Maternal cys lycopene blood levels"

126 .
127 . sum maternaltotallycopenemcgl

        Variable |        Obs        Mean    Std. dev.       Min        Max
    -------------+---------------------------------------------------------
    maternalto.. |        225    545.4308    257.6176      25.81    1521.69

128 . sktest maternaltotallycopenemcgl

    Skewness and kurtosis tests for normality
                                                                       ----- J
    > oint test -----
                     Variable |       Obs   Pr(skewness)   Pr(kurtosis)   Adj chi
    > 2(2)  Prob>chi2
    --------------------------+--------------------------------------------------
    > ---------------
    maternaltotallycopenemcgl |       225         0.0040         0.4360
    > 8.19
    >          0.0167

129 . hist maternalcislycopenemcgl
    (bin=15, start=15.48, width=49.942667)

130 . lab var maternaltotallycopenemcgl "Maternal total lycopene blood levels"

131 .
132 . sum maternalacarotenemcgl

        Variable |        Obs        Mean    Std. dev.       Min        Max
    -------------+---------------------------------------------------------
    maternalac~l |        225    66.48474     98.6931       2.38    1022.34
```

```
133 . sktest maternalacarotenemcgl

    Skewness and kurtosis tests for normality
                                                                   ----- Joint
    >  test -----
                 Variable |       Obs   Pr(skewness)   Pr(kurtosis)   Adj chi2(2)
    >   Prob>chi2
    ----------------------+----------------------------------------------------------
    > -----------
    maternalacarotenemcgl |       225         0.0000         0.0000        205.68
    >      0.0000

134 . hist maternalacarotenemcgl
    (bin=15, start=2.38, width=67.997333)

135 . lab var maternalacarotenemcgl "Maternal a-carotene blood levels"

136 .
137 . sum maternaltransbcarotenemcgl

        Variable |        Obs        Mean    Std. dev.       Min        Max
    -------------+---------------------------------------------------------
    maternaltr.. |        225    224.0389    252.7003       7.08    2792.48

138 . sktest maternaltransbcarotenemcgl

    Skewness and kurtosis tests for normality
                                                                        -----
    > Joint test -----
                      Variable |       Obs   Pr(skewness)   Pr(kurtosis)   Adj ch
    > i2(2)  Prob>chi2
    ---------------------------+-----------------------------------------------------
    > ----------------
    maternaltransbcarotenemcgl |       225         0.0000         0.0000         2
    > 00.46
    >          0.0000
```

```
139 . hist maternaltransbcarotenemcgl
    (bin=15, start=7.08, width=185.69333)

140 . lab var maternaltransbcarotenemcgl "Maternal trans b-carotene blood levels"

141 .
142 . sum maternalcisbcarotenemcgl

        Variable |        Obs        Mean    Std. dev.       Min        Max
    -------------+---------------------------------------------------------
    maternalci.. |        222    18.31692    19.97374       1.41      210.6

143 . sktest maternalcisbcarotenemcgl

    Skewness and kurtosis tests for normality
                                                                    ----- Jo
    > int test -----
                    Variable |       Obs   Pr(skewness)   Pr(kurtosis)   Adj chi2
    > (2)  Prob>chi2
    -------------------------+----------------------------------------------------
    > --------------
    maternalcisbcarotenemcgl |       222         0.0000         0.0000        183
    > .00
    >         0.0000

144 . hist maternalcisbcarotenemcgl
    (bin=14, start=1.41, width=14.942143)

145 . lab var maternalcisbcarotenemcgl "Maternal cis b-carotene blood levels"

146 .
147 . sum maternaltotalbcarotenemcgl

        Variable |        Obs        Mean    Std. dev.       Min        Max
    -------------+---------------------------------------------------------
    maternalto.. |        225    242.1113    272.1941       7.08    3003.08
```

```
148 . sktest maternaltotalbcarotenemcgl

    Skewness and kurtosis tests for normality
                                                                        ─────
    > Joint test ─────
                      Variable │       Obs   Pr(skewness)   Pr(kurtosis)   Adj ch
    > i2(2)  Prob>chi2
    ───────────────────────────┼──────────────────────────────────────────────────
    > ────────────────
    maternaltotalbcarotenemcgl │       225         0.0000         0.0000        1
    > 99.78
    >          0.0000

149 . hist maternaltotalbcarotenemcgl
    (bin=15, start=7.08, width=199.73333)

150 . lab var maternaltotalbcarotenemcgl "Maternal total b-carotene blood levels"

151 .
152 . *generating descriptive statistics for maternal demographics
153 .
154 . sktest age

    Skewness and kurtosis tests for normality
                                                          ───── Joint test ───
    > ──
        Variable │       Obs   Pr(skewness)   Pr(kurtosis)   Adj chi2(2)  Prob>ch
    > i2
    ─────────────┼────────────────────────────────────────────────────────────────
    > ──
             age │       486         0.4039         0.0001         14.25     0.00
    > 08

155 . sktest bmi

    Skewness and kurtosis tests for normality
                                                          ───── Joint test ───
    > ──
        Variable │       Obs   Pr(skewness)   Pr(kurtosis)   Adj chi2(2)  Prob>ch
    > i2
    ─────────────┼────────────────────────────────────────────────────────────────
    > ──
             bmi │       460         0.0000         0.0001         57.75     0.00
    > 00
```

```
156 . sktest ga

    Skewness and kurtosis tests for normality
                                                            ───── Joint test ───
    > ──
        Variable │       Obs   Pr(skewness)   Pr(kurtosis)   Adj chi2(2)  Prob>ch
    > i2
    ─────────────┼─────────────────────────────────────────────────────────────
    > ──
              ga │       481         0.0000         0.0000        170.04     0.00
    > 00
```

```
157 . tabstat age bmi ga, by(htncat) statistic(median n p25 p75)

    Summary statistics: p50, N, p25, p75
    Group variable: htncategoryaccaha (HDP Status)
```

htncategoryaccaha	age	bmi	ga
NT	30	27.34	39.3
	268	255	267
	25	23.39	38.4
	33	31.76	40.2
CH	30	26.82	39.4
	61	61	60
	25	22.39	38.25
	33	33.2	40.35
GH	30	27.96	39.2
	102	95	99
	25	22.86	38
	34	32.4	40.3
PE	28	34.18	37
	55	49	55
	23	25.73	34.1
	34	38.77	38.6
Total	30	27.615	39.2
	486	460	481
	25	23.375	37.6
	33	33.09	40.1

```
158 . kwallis age, by(htncat)

    Kruskal—Wallis equality-of-populations rank test
```

htncat~a	Obs	Rank sum
NT	268	65324.00
CH	61	15251.50
GH	102	25462.50
PE	55	12303.00

```
      chi2(3) =  1.421
         Prob = 0.7006

      chi2(3) with ties =  1.426
                   Prob = 0.6996

159 . kwallis bmi, by(htncat)

    Kruskal—Wallis equality-of-populations rank test
```

htncat~a	Obs	Rank sum
NT	255	56311.00
CH	61	12970.00
GH	95	21905.50
PE	49	14843.50

```
      chi2(3) = 16.999
         Prob = 0.0007

      chi2(3) with ties = 16.999
                   Prob = 0.0007
```

```
160 . tab race htncat, chi2 column

  +-------------------+
  | Key               |
  |-------------------|
  |     frequency     |
  | column percentage |
  +-------------------+

                      |                 HDP Status
        Maternal Race |        NT         CH         GH         PE |      Tota
> l
----------------------+--------------------------------------------+----------
> -
                White |       180         46         73         37 |        33
> 6
                      |     66.91      75.41      71.57      67.27 |      68.9
> 9
----------------------+--------------------------------------------+----------
> -
     African American |        40          6          9          8 |         6
> 3
                      |     14.87       9.84       8.82      14.55 |      12.9
> 4
----------------------+--------------------------------------------+----------
> -
             Hispanic |        22          2          7          3 |         3
> 4
                      |      8.18       3.28       6.86       5.45 |       6.9
> 8
----------------------+--------------------------------------------+----------
> -
Asian or Pacific Isla |         6          3          1          2 |         1
> 2
                      |      2.23       4.92       0.98       3.64 |       2.4
> 6
----------------------+--------------------------------------------+----------
> -
      American Indian |         0          1          0          0 |
> 1
                      |      0.00       1.64       0.00       0.00 |       0.2
> 1
----------------------+--------------------------------------------+----------
> -
        Other/Unknown |        21          3         12          5 |         4
> 1
```

	**7.81**	**4.92**	**11.76**	**9.09**	**8.4**
**> 2**					
> —					
Total	**269**	**61**	**102**	**55**	**48**
**> 7**					
	**100.00**	**100.00**	**100.00**	**100.00**	**100.0**
**> 0**					

Pearson chi2(**15**) = **17.4098** Pr = **0.295**

161 . tab par htncat, chi2 column

Key
frequency<br>*column percentage*

Maternal Parity Bins	HDP Status NT	CH	GH	PE	Total
Nulliparous	**31**<br>**11.48**	**4**<br>**6.56**	**11**<br>**10.78**	**14**<br>**25.45**	**60**<br>**12.30**
Primiparous	**83**<br>**30.74**	**24**<br>**39.34**	**37**<br>**36.27**	**20**<br>**36.36**	**164**<br>**33.61**
Multiparous	**156**<br>**57.78**	**33**<br>**54.10**	**54**<br>**52.94**	**21**<br>**38.18**	**264**<br>**54.10**
Total	**270**<br>**100.00**	**61**<br>**100.00**	**102**<br>**100.00**	**55**<br>**100.00**	**488**<br>**100.00**

Pearson chi2(**6**) = **14.5889** Pr = **0.024**

```
162 . tab maternaldiab htncat, chi2 column
```

Key
frequency<br>*column percentage*

Maternal Diabetes Status	HDP Status NT	CH	GH	PE	Total
No Diabetes	**236**<br>**87.41**	**57**<br>**93.44**	**92**<br>**90.20**	**44**<br>**80.00**	**429**<br>**87.91**
Diabetes	**34**<br>**12.59**	**4**<br>**6.56**	**10**<br>**9.80**	**11**<br>**20.00**	**59**<br>**12.09**
Total	**270**<br>**100.00**	**61**<br>**100.00**	**102**<br>**100.00**	**55**<br>**100.00**	**488**<br>**100.00**

Pearson chi2(**3**) = **5.5603** Pr = **0.135**

```
163 . tab smoking htncat, chi2 column
```

Key
frequency<br>*column percentage*

Maternal Smoking Status	HDP Status NT	CH	GH	PE	Total
Never	**210**<br>**77.78**	**51**<br>**83.61**	**78**<br>**76.47**	**38**<br>**69.09**	**377**<br>**77.25**
Current	**28**<br>**10.37**	**5**<br>**8.20**	**7**<br>**6.86**	**5**<br>**9.09**	**45**<br>**9.22**
Former	**32**<br>**11.85**	**5**<br>**8.20**	**17**<br>**16.67**	**12**<br>**21.82**	**66**<br>**13.52**

```
     Total |       270         61        102         55 |       488 
           |    100.00     100.00     100.00     100.00 |    100.00 

          Pearson chi2(6) =   7.2638   Pr = 0.297

164 . 
165 . *log transforming for exploratory regression adjustments
166 . 
167 . gen log_acar=log(acar)

168 . gen log_bcar=log(bcar)

169 . gen log_bcryp=log(bcryp)

170 . gen log_lut=log(lut)

171 . gen log_lyco=log(lyco)
    (1 missing value generated)

172 . 
173 . gen log_lutzea=log(maternalluteinzeaxanthinmc)
    (263 missing values generated)

174 . gen log_bcrypto=log(maternalbcryptoxanthinmcgl)
    (263 missing values generated)

175 . gen log_translyco=log(maternaltranslycopenemcgl)
    (263 missing values generated)

176 . gen log_cislyco=log(maternalcislycopenemcgl)
    (263 missing values generated)

177 . gen log_totallyco=log(maternaltotallycopenemcgl)
    (263 missing values generated)
```

```
178 . gen log_acarot=log(maternalacarotenemcgl)
    (263 missing values generated)

179 . gen log_transbcarot=log(maternaltransbcarotenemcgl)
    (263 missing values generated)

180 . gen log_cisbcarot=log(maternalcisbcarotenemcgl)
    (266 missing values generated)

181 . gen log_totalbcarot=log(maternaltotalbcarotenemcgl)
    (263 missing values generated)

182 .
183 . *Performing exploratory regression adjustments
184 .
185 . mlogit htncategoryaccaha log_acar i.par bmi, vce(robust) rrr

    Iteration 0:  Log pseudolikelihood = -533.26118
    Iteration 1:  Log pseudolikelihood = -519.85054
    Iteration 2:  Log pseudolikelihood = -517.95585
    Iteration 3:  Log pseudolikelihood = -517.94794
    Iteration 4:  Log pseudolikelihood = -517.94794

    Multinomial logistic regression                         Number of obs =    46
    > 0
                                                            Wald chi2(12) =  30.5
    > 3
                                                            Prob > chi2   = 0.002
    > 3
    Log pseudolikelihood = -517.94794                       Pseudo R2     = 0.028
    > 7

    ----------------------------------------------------------------------------
    > -
                 |               Robust
    htncategor~a |        RRR   std. err.      z    P>|z|     [95% conf. interval
    > ]
    -------------+--------------------------------------------------------------
    > -
    NT           |  (base outcome)
    -------------+--------------------------------------------------------------
    > -
    CH           |
        log_acar |   1.046728   .1186284     0.40   0.687     .8382329    1.30708
    > 1
                 |
```

```
         par |
 Primiparous |   2.010763   1.178046     1.19   0.233      .637775    6.33948
> 9
 Multiparous |   1.379064   .7810604     0.57   0.570     .4544537    4.18484
> 5
             |
         bmi |   1.002751   .0245639     0.11   0.911     .9557444     1.0520
> 7
       _cons |   .1097016   .1343319    -1.80   0.071     .0099517     1.2092
> 8
-------------+----------------------------------------------------------------
> -
GH           |
    log_acar |   .9423632    .091133    -0.61   0.539     .7796528    1.13903
> 1
             |
         par |
 Primiparous |   1.339334   .5860585     0.67   0.504     .5681001     3.1575
> 7
 Multiparous |   .9881236   .4208363    -0.03   0.978     .4288341    2.27684
> 4
             |
         bmi |   1.008171    .016947     0.48   0.628     .9754971     1.0419
> 4
       _cons |   .3843762    .313863    -1.17   0.242     .0775723    1.90461
> 2
-------------+----------------------------------------------------------------
> -
PE           |
    log_acar |   .8709484    .114985    -1.05   0.295     .6723795    1.12815
> 9
             |
         par |
 Primiparous |   .6339383   .3024359    -0.96   0.339      .248863    1.61485
> 5
 Multiparous |   .2966683   .1373688    -2.62   0.009     .1197105    .735207
> 6
             |
         bmi |   1.086619   .0230365     3.92   0.000     1.042394    1.13272
> 1
       _cons |   .0734285   .0810402    -2.37   0.018     .0084416    .638708
> 1
-------------+----------------------------------------------------------------
> -
Note: _cons estimates baseline relative risk for each outcome.
```

```
186 . mlogit htncategoryaccaha log_bcar i.par bmi, vce(robust) rrr

    Iteration 0:  Log pseudolikelihood = -533.26118
    Iteration 1:  Log pseudolikelihood = -519.89358
    Iteration 2:  Log pseudolikelihood =  -517.9978
    Iteration 3:  Log pseudolikelihood =  -517.9895
    Iteration 4:  Log pseudolikelihood =  -517.9895

    Multinomial logistic regression                        Number of obs =    46
    > 0
                                                           Wald chi2(12) =  30.2
    > 7
                                                           Prob > chi2   = 0.002
    > 5
    Log pseudolikelihood = -517.9895                       Pseudo R2     = 0.028
    > 6

    ─────────────┬──────────────────────────────────────────────────────────────
    > ─
                 │              Robust
    htncategor~a │        RRR   std. err.      z    P>|z|     [95% conf. interval
    > ]
    ─────────────┼──────────────────────────────────────────────────────────────
    > ─
    NT           │  (base outcome)
    ─────────────┼──────────────────────────────────────────────────────────────
    > ─
    CH           │
        log_bcar │   .9644702   .1928146    -0.18   0.856     .6518064    1.42711
    > 5
                 │
             par │
     Primiparous │   2.016019   1.187626     1.19   0.234     .6354133    6.39635
    > 9
     Multiparous │   1.396639   .8025268     0.58   0.561      .452869    4.30720
    > 5
                 │
             bmi │   1.002438   .0242224     0.10   0.920     .9560699    1.05105
    > 6
           _cons │   .1963666   .3889679    -0.82   0.411     .0040456    9.53122
    > 9
    ─────────────┼──────────────────────────────────────────────────────────────
    > ─
    GH           │
        log_bcar │   .8521731   .1372032    -0.99   0.320     .6215571    1.16835
    > 4
```

```
             |
         par |
 Primiparous |   1.290412   .5602381     0.59   0.557     .5510302    3.02190
> 9
 Multiparous |   .9291516   .3917644    -0.17   0.862     .4066201    2.12316
> 8
             |
         bmi |   1.010639   .0171536     0.62   0.533     .9775715    1.04482
> 5
       _cons |   1.013881   1.466688     0.01   0.992     .0595158    17.2719
> 7
-------------+----------------------------------------------------------------
> -
PE           |
    log_bcar |   .7836136   .1823451    -1.05   0.295     .4966259    1.23644
> 4
             |
         par |
 Primiparous |   .6082044   .2942722    -1.03   0.304     .2356168    1.56997
> 6
 Multiparous |   .2711769   .1276576    -2.77   0.006      .107782    .682274
> 5
             |
         bmi |   1.090855   .0234823     4.04   0.000     1.045787    1.13786
> 4
       _cons |   .2409684    .498966    -0.69   0.492      .004163    13.9481
> 8
------------------------------------------------------------------------------
> -
Note: _cons estimates baseline relative risk for each outcome.

187 . mlogit htncategoryaccaha log_bcryp i.par bmi, vce(robust) rrr

Iteration 0:  Log pseudolikelihood = -533.26118
Iteration 1:  Log pseudolikelihood = -520.22026
Iteration 2:  Log pseudolikelihood = -518.35381
Iteration 3:  Log pseudolikelihood = -518.34677
Iteration 4:  Log pseudolikelihood = -518.34677
```

```
Multinomial logistic regression                        Number of obs =    46
> 0
                                                       Wald chi2(12) =  30.8
> 6
                                                       Prob > chi2   = 0.002
> 1
Log pseudolikelihood = -518.34677                      Pseudo R2     = 0.028
> 0

─────────────┬──────────────────────────────────────────────────────────────
> ─
             │               Robust
htncategor~a │        RRR   std. err.      z    P>|z|     [95% conf. interval
> ]
─────────────┼──────────────────────────────────────────────────────────────
> ─
NT           │  (base outcome)
─────────────┼──────────────────────────────────────────────────────────────
> ─
CH           │
   log_bcryp │   .9080388   .1390416    -0.63   0.529     .6726139    1.22586
> 6
             │
         par │
Primiparous  │    2.02633   1.185926     1.21   0.228     .6434837    6.38091
> 1
Multiparous  │    1.39382   .7978227     0.58   0.562     .4539194    4.27990
> 9
             │
         bmi │    1.00146   .0243872     0.06   0.952     .9547847    1.05041
> 7
       _cons │     .23351   .2721897    -1.25   0.212     .0237743    2.29352
> 7
─────────────┼──────────────────────────────────────────────────────────────
> ─
GH           │
   log_bcryp │     .88471   .1219648    -0.89   0.374     .6752359    1.15916
> 8
             │
         par │
Primiparous  │   1.319703   .5758074     0.64   0.525     .5611556    3.10362
> 3
Multiparous  │    .946891   .3992795    -0.13   0.897     .4143528    2.16386
> 3
             │
         bmi │   1.008142   .0169939     0.48   0.630     .9753791    1.04200
```

```
> 6
       _cons │   .4925258    .444346    -0.78   0.432     .0840414    2.88645
> 3
─────────────┼────────────────────────────────────────────────────────────────
> -
PE           │
   log_bcryp │   .9879661    .180416    -0.07   0.947     .6907185    1.41313
> 3
             │
         par │
Primiparous  │   .6256204   .3018794    -0.97   0.331     .2429866    1.61079
> 2
Multiparous  │   .2858506   .1330027    -2.69   0.007      .114838    .711528
> 8
             │
         bmi │   1.086963   .0231394     3.92   0.000     1.042544    1.13327
> 5
       _cons │   .0351397   .0435506    -2.70   0.007     .0030964     .39878
> 6
─────────────┴────────────────────────────────────────────────────────────────
> -
Note: _cons estimates baseline relative risk for each outcome.

188 . mlogit htncategoryaccaha log_lut i.par bmi, vce(robust) rrr

Iteration 0:  Log pseudolikelihood = -533.26118
Iteration 1:  Log pseudolikelihood = -520.08401
Iteration 2:  Log pseudolikelihood = -518.20144
Iteration 3:  Log pseudolikelihood = -518.19435
Iteration 4:  Log pseudolikelihood = -518.19435

Multinomial logistic regression                        Number of obs =    46
> 0
                                                       Wald chi2(12) =  32.1
> 7
                                                       Prob > chi2   = 0.001
> 3
Log pseudolikelihood = -518.19435                      Pseudo R2     = 0.028
> 3
```

htncategor~a	RRR	Robust std. err.	z	P>|z|	[95% conf. interval	>]
> −						
**NT**	(base outcome)					
> −						
**CH**						
log_lut	**.9556155**	**.1740972**	**−0.25**	**0.803**	**.6686648**	**1.36570**
**> 8**						
par						
Primiparous	**2.02477**	**1.187971**	**1.20**	**0.229**	**.6411499**	**6.39428**
**> 5**						
Multiparous	**1.40014**	**.8019259**	**0.59**	**0.557**	**.455668**	**4.30223**
**> 9**						
bmi	**1.002342**	**.0243071**	**0.10**	**0.923**	**.9558156**	**1.05113**
**> 3**						
_cons	**.2061143**	**.3657567**	**−0.89**	**0.373**	**.0063624**	**6.67719**
**> 3**						
> −						
**GH**						
log_lut	**.8391947**	**.1407075**	**−1.05**	**0.296**	**.6041463**	**1.16569**
**> 1**						
par						
Primiparous	**1.312968**	**.5683266**	**0.63**	**0.529**	**.5620901**	**3.06691**
**> 8**						
Multiparous	**.9398161**	**.3945914**	**−0.15**	**0.882**	**.4127216**	**2.14007**
**> 3**						
bmi	**1.009868**	**.0170317**	**0.58**	**0.560**	**.9770321**	**1.04380**
**> 7**						
_cons	**1.039187**	**1.48886**	**0.03**	**0.979**	**.062685**	**17.2275**
**> 6**						
> −						
**PE**						
log_lut	**1.034501**	**.2525008**	**0.14**	**0.889**	**.6411653**	**1.66913**
**> 7**						

```
         par |
 Primiparous |   .6273313     .30327    -0.96   0.335     .2432213    1.61805
> 1
 Multiparous |   .2874736   .1356712    -2.64   0.008     .1139933    .724964
> 3
             |
         bmi |   1.086714   .0231406     3.91   0.000     1.042293    1.13302
> 9
       _cons |   .0255186   .0516278    -1.81   0.070     .0004839    1.34573
> 2
-------------+----------------------------------------------------------------
> -
Note: _cons estimates baseline relative risk for each outcome.

189 . mlogit htncategoryaccaha log_lyco i.par bmi, vce(robust) rrr

Iteration 0:  Log pseudolikelihood = -532.67034
Iteration 1:  Log pseudolikelihood = -519.93944
Iteration 2:  Log pseudolikelihood = -518.08564
Iteration 3:  Log pseudolikelihood = -518.07853
Iteration 4:  Log pseudolikelihood = -518.07853

Multinomial logistic regression                        Number of obs =    45
> 9
                                                       Wald chi2(12) =  29.5
> 8
                                                       Prob > chi2   = 0.003
> 2
Log pseudolikelihood = -518.07853                      Pseudo R2     = 0.027
> 4

------------------------------------------------------------------------------
> -
             |               Robust
htncategor~a |        RRR   std. err.      z    P>|z|     [95% conf. interval
> ]
-------------+----------------------------------------------------------------
> -
NT           |  (base outcome)
-------------+----------------------------------------------------------------
> -
CH           |
    log_lyco |    1.13771   .2752325     0.53   0.594     .7081258      1.827
> 9
             |
         par |
```

```
Primiparous  |   2.044241   1.204287     1.21   0.225     .6442864    6.48612
> 2
Multiparous  |   1.443861   .8327721     0.64   0.524     .4662085     4.4716
> 8
             |
         bmi |   1.001892   .0243747     0.08   0.938     .9552392    1.05082
> 3
       _cons |   .0484163   .1062723    -1.38   0.168     .0006556    3.57564
> 2
-------------+----------------------------------------------------------------
> -
GH           |
    log_lyco |   1.018396   .1827644     0.10   0.919     .7164028    1.44769
> 1
             |
         par |
Primiparous  |   1.322915   .5788656     0.64   0.522     .5611428    3.11882
> 3
Multiparous  |   .9688764   .4101468    -0.07   0.940     .4226062    2.22126
> 7
             |
         bmi |   1.008617   .0168674     0.51   0.608     .9760938    1.04222
> 5
       _cons |   .2328311   .3990352    -0.85   0.395      .008095    6.69675
> 4
-------------+----------------------------------------------------------------
> -
PE           |
    log_lyco |   1.093706   .2201493     0.44   0.656     .7371643    1.62269
> 5
             |
         par |
Primiparous  |    .631224   .3054201    -0.95   0.342     .2445267    1.62944
> 8
Multiparous  |    .291555   .1361975    -2.64   0.008     .1167048    .728370
> 3
             |
         bmi |   1.086771   .0230179     3.93   0.000      1.04258    1.13283
> 4
       _cons |   .0154956   .0288112    -2.24   0.025     .0004051    .592732
> 7
------------------------------------------------------------------------------
> -
Note: _cons estimates baseline relative risk for each outcome.
```

```
190 .
191 . mlogit htncategoryaccaha log_lutzea i.par bmi, vce(robust) rrr

    Iteration 0:  Log pseudolikelihood = -226.42425
    Iteration 1:  Log pseudolikelihood = -219.02664
    Iteration 2:  Log pseudolikelihood = -217.04929
    Iteration 3:  Log pseudolikelihood = -216.99382
    Iteration 4:  Log pseudolikelihood = -216.99369
    Iteration 5:  Log pseudolikelihood = -216.99369

    Multinomial logistic regression                        Number of obs =    21
    > 0
                                                           Wald chi2(12) =  19.6
    > 9
                                                           Prob > chi2   = 0.073
    > 2
    Log pseudolikelihood = -216.99369                      Pseudo R2     = 0.041
    > 6

    ─────────────┬─────────────────────────────────────────────────────────────
    > ─
                 │               Robust
    htncategor~a │        RRR   std. err.      z    P>|z|     [95% conf. interval
    > ]
    ─────────────┼─────────────────────────────────────────────────────────────
    > ─
    NT           │  (base outcome)
    ─────────────┼─────────────────────────────────────────────────────────────
    > ─
    CH           │
      log_lutzea │   .6805182   .3014261    -0.87   0.385     .2856352    1.62131
    > 6
                 │
             par │
    Primiparous  │    .639097   .5831394    -0.49   0.624     .1068794    3.82154
    > 9
    Multiparous  │   .4549045   .4064363    -0.88   0.378     .0789607    2.62077
    > 5
                 │
             bmi │   .9489034    .037518    -1.33   0.185     .8781464    1.02536
    > 2
           _cons │      13.88   39.66783     0.92   0.357     .0512542    3758.80
    > 2
    ─────────────┼─────────────────────────────────────────────────────────────
    > ─
    GH           │
```

```
  log_lutzea │   1.311888   .5227942     0.68   0.496     .6007427     2.8648
> 7
             │
         par │
Primiparous  │   1.760734   2.050802     0.49   0.627     .1795822    17.2633
> 2
Multiparous  │   1.304463   1.513077     0.23   0.819     .1343084    12.6695
> 3
             │
         bmi │   1.027742   .0289267     0.97   0.331     .9725818     1.0860
> 3
       _cons │   .0298431   .0799526    -1.31   0.190     .0001565    5.69241
> 6
─────────────┼────────────────────────────────────────────────────────────────
> ─
PE           │
  log_lutzea │   .6745018    .445541    -0.60   0.551     .1848091    2.46174
> 4
             │
         par │
Primiparous  │   .1219239   .1156102    -2.22   0.026     .0190093    .782009
> 3
Multiparous  │   .0483368   .0471404    -3.11   0.002     .0071474    .326896
> 2
             │
         bmi │   1.099968   .0557894     1.88   0.060     .9958823    1.21493
> 3
       _cons │   .4207079   1.760329    -0.21   0.836     .0001154    1533.14
> 9
─────────────┴────────────────────────────────────────────────────────────────
> ─
Note: _cons estimates baseline relative risk for each outcome.

192 . mlogit htncategoryaccaha log_bcrypto i.par bmi, vce(robust) rrr

Iteration 0:  Log pseudolikelihood = -226.42425
Iteration 1:  Log pseudolikelihood = -219.13239
Iteration 2:  Log pseudolikelihood = -217.22786
Iteration 3:  Log pseudolikelihood = -217.17477
Iteration 4:  Log pseudolikelihood = -217.17468
Iteration 5:  Log pseudolikelihood = -217.17468
```

```
Multinomial logistic regression                         Number of obs =    21
> 0
                                                        Wald chi2(12) =  24.8
> 8
                                                        Prob > chi2   = 0.015
> 4
Log pseudolikelihood = -217.17468                       Pseudo R2     = 0.040
> 9

─────────────┬────────────────────────────────────────────────────────────────
> ─
             │              Robust
htncategor~a │        RRR   std. err.      z    P>|z|     [95% conf. interval
> ]
─────────────┼────────────────────────────────────────────────────────────────
> ─
NT           │  (base outcome)
─────────────┼────────────────────────────────────────────────────────────────
> ─
CH           │
 log_bcrypto │   .9421046    .314707    -0.18   0.858     .4895061    1.81317
> 7
             │
         par │
Primiparous  │   .6056183    .560242    -0.54   0.588     .0988039    3.71213
> 5
Multiparous  │   .4374558   .3948233    -0.92   0.360     .0745911    2.56555
> 5
             │
         bmi │   .9542155    .036687    -1.22   0.223     .8849527    1.02889
> 9
       _cons │   2.147242   4.576207     0.36   0.720     .0329457    139.946
> 9
─────────────┼────────────────────────────────────────────────────────────────
> ─
GH           │
 log_bcrypto │   1.170721   .3656324     0.50   0.614     .6347626    2.15921
> 3
             │
         par │
Primiparous  │    1.81266   2.091399     0.52   0.606     .1888961    17.3944
> 1
Multiparous  │   1.321353   1.515411     0.24   0.808     .1395747    12.5092
> 4
             │
         bmi │   1.024973   .0281035     0.90   0.368     .9713451    1.08156
```

```
> 2
       _cons |    .063376   .1181618    -1.48   0.139     .0016402    2.44879
> 5
-------------+----------------------------------------------------------------
> -
PE           |
 log_bcrypto |   .6329262   .2753334    -1.05   0.293     .2698151    1.48470
> 4
             |
         par |
Primiparous  |   .1221187   .1169181    -2.20   0.028     .0186995    .797505
> 9
Multiparous  |   .0508235   .0500535    -3.03   0.002     .0073749    .350243
> 4
             |
         bmi |   1.099651   .0577858     1.81   0.071     .9920306    1.21894
> 8
       _cons |   .4395399   1.344757    -0.27   0.788     .0010934    176.693
> 3
-------------+----------------------------------------------------------------
> -
Note: _cons estimates baseline relative risk for each outcome.
```

193 . mlogit htncategoryaccaha log_translyco i.par bmi, vce(robust) rrr

```
Iteration 0:  Log pseudolikelihood = -226.42425
Iteration 1:  Log pseudolikelihood = -219.28968
Iteration 2:  Log pseudolikelihood =  -217.3586
Iteration 3:  Log pseudolikelihood = -217.30557
Iteration 4:  Log pseudolikelihood = -217.30548
Iteration 5:  Log pseudolikelihood = -217.30548

Multinomial logistic regression                        Number of obs =    21
> 0
                                                       Wald chi2(12) =  18.9
> 1
                                                       Prob > chi2   = 0.090
> 7
Log pseudolikelihood = -217.30548                      Pseudo R2     = 0.040
> 3
```

```
──────────────┬─────────────────────────────────────────────────────────
> ──
              │               Robust
htncategory~a │        RRR   std. err.      z    P>|z|     [95% conf. interva
> l]
──────────────┼─────────────────────────────────────────────────────────
> ──
NT            │  (base outcome)
──────────────┼─────────────────────────────────────────────────────────
> ──
CH            │
log_translyco │   1.066336   .3419323     0.20   0.841     .5687839    1.9991
> 31
              │
          par │
 Primiparous  │   .5959152   .5610294    -0.55   0.582     .0941485    3.7718
> 59
 Multiparous  │   .4266867   .3943442    -0.92   0.357     .0697313    2.6109
> 01
              │
          bmi │   .9552258   .0369733    -1.18   0.237       .88544    1.0305
> 12
        _cons │   1.122184   2.227185     0.06   0.954     .0229454    54.882
> 42
──────────────┼─────────────────────────────────────────────────────────
> ──
GH            │
log_translyco │   1.335151   .4333159     0.89   0.373     .7067689    2.5222
> 21
              │
          par │
 Primiparous  │   1.813513   2.111586     0.51   0.609     .1851031    17.767
> 55
 Multiparous  │   1.277148    1.47527     0.21   0.832     .1327376     12.28
> 82
              │
          bmi │   1.024335    .028022     0.88   0.379      .970859    1.0807
> 56
        _cons │   .0280648   .0636354    -1.58   0.115     .0003297    2.3889
> 95
──────────────┼─────────────────────────────────────────────────────────
> ──
PE            │
log_translyco │   .8286835   .3746249    -0.42   0.678      .341652    2.0099
> 88
              │
```

```
          par |
  Primiparous |    .109854   .1043423    -2.33   0.020     .0170735    .70682
> 19
  Multiparous |   .0459754   .0436935    -3.24   0.001     .0071379    .29612
> 76
              |
          bmi |   1.108564   .0537087     2.13   0.033      1.00814    1.2189
> 91
        _cons |   .1256309   .3461012    -0.75   0.451     .0005677    27.802
> 05
--------------------------------------------------------------------------------
> --
Note: _cons estimates baseline relative risk for each outcome.

194 . mlogit htncategoryaccaha log_cislyco i.par bmi, vce(robust) rrr

Iteration 0:  Log pseudolikelihood = -226.42425
Iteration 1:  Log pseudolikelihood = -219.33748
Iteration 2:  Log pseudolikelihood = -217.45312
Iteration 3:  Log pseudolikelihood = -217.40229
Iteration 4:  Log pseudolikelihood = -217.40217
Iteration 5:  Log pseudolikelihood = -217.40217

Multinomial logistic regression                         Number of obs =    21
> 0
                                                        Wald chi2(12) =  19.0
> 2
                                                        Prob > chi2   = 0.088
> 0
Log pseudolikelihood = -217.40217                       Pseudo R2     = 0.039
> 8

--------------------------------------------------------------------------------
> -
             |               Robust
htncategor~a |        RRR   std. err.      z    P>|z|     [95% conf. interval
> ]
--------------------------------------------------------------------------------
> -
NT           |  (base outcome)
--------------------------------------------------------------------------------
> -
CH           |
 log_cislyco |   1.185962   .3649639     0.55   0.579     .6488213    2.16778
> 7
             |
```

```
         par |
 Primiparous |   .5857409   .5540798    -0.57   0.572     .0917306    3.74021
> 9
 Multiparous |   .4146706   .3847524    -0.95   0.343     .0672846     2.5555
> 9
             |
         bmi |   .9565904   .0366394    -1.16   0.247     .8874078    1.03116
> 7
       _cons |   .6191262   1.181218    -0.25   0.802      .014716    26.0476
> 7
-------------+----------------------------------------------------------------
> -
GH           |
 log_cislyco |   1.345341    .470128     0.85   0.396     .6782361    2.66860
> 2
             |
         par |
 Primiparous |   1.789303   2.084772     0.50   0.618     .1823572    17.5567
> 9
 Multiparous |   1.262663   1.460025     0.20   0.840     .1309297     12.176
> 9
             |
         bmi |   1.025859   .0280978     0.93   0.351     .9722404    1.08243
> 4
       _cons |   .0267194   .0636329    -1.52   0.128      .000251    2.84436
> 8
-------------+----------------------------------------------------------------
> -
PE           |
 log_cislyco |   1.056881   .5129329     0.11   0.909     .4082394    2.73613
> 3
             |
         par |
 Primiparous |   .1072664   .1024355    -2.34   0.019     .0165043    .697156
> 9
 Multiparous |    .043977   .0422804    -3.25   0.001     .0066813    .289459
> 5
             |
         bmi |   1.109288   .0540618     2.13   0.033     1.008232    1.22047
> 2
       _cons |   .0337324   .1030772    -1.11   0.267     .0000845    13.4614
> 5
-------------+----------------------------------------------------------------
> -
Note: _cons estimates baseline relative risk for each outcome.
```

```
195 . mlogit htncategoryaccaha log_totallyco i.par bmi, vce(robust) rrr

    Iteration 0:  Log pseudolikelihood = -226.42425
    Iteration 1:  Log pseudolikelihood = -219.31793
    Iteration 2:  Log pseudolikelihood = -217.41353
    Iteration 3:  Log pseudolikelihood = -217.36173
    Iteration 4:  Log pseudolikelihood = -217.36165
    Iteration 5:  Log pseudolikelihood = -217.36165

    Multinomial logistic regression                        Number of obs =    21
    > 0
                                                           Wald chi2(12) =  18.9
    > 2
                                                           Prob > chi2   = 0.090
    > 4
    Log pseudolikelihood = -217.36165                      Pseudo R2     = 0.040
    > 0

    ──────────────┬──────────────────────────────────────────────────────────────
    > ──
                  │               Robust
    htncategory~a │        RRR   std. err.      z    P>|z|     [95% conf. interva
    > l]
    ──────────────┼──────────────────────────────────────────────────────────────
    > ──
    NT            │  (base outcome)
    ──────────────┼──────────────────────────────────────────────────────────────
    > ──
    CH            │
    log_totallyco │   1.131335   .3612771     0.39   0.699     .6050219    2.1154
    > 92
                  │
              par │
     Primiparous  │   .5910427   .5579007    -0.56   0.577     .0929282    3.7591
    > 56
     Multiparous  │   .4206028   .3894949    -0.94   0.350     .0684898    2.5829
    > 66
                  │
              bmi │   .9558277   .0367854    -1.17   0.240     .8863817    1.0307
    > 15
            _cons │   .7403018   1.579223    -0.14   0.888     .0113139    48.440
    > 11
    ──────────────┼──────────────────────────────────────────────────────────────
    > ──
    GH            │
    log_totallyco │   1.350589   .4649993     0.87   0.383     .6877964    2.6520
```

```
> 79
               |
           par |
  Primiparous  |   1.798052   2.094774     0.50   0.615     .1832869    17.638
> 96
  Multiparous  |   1.267825    1.46541     0.21   0.837     .1315836    12.215
> 66
               |
           bmi |   1.025065   .0280484     0.90   0.366     .9715392     1.081
> 54
         _cons |   .0213293   .0546448    -1.50   0.133     .0001407    3.2338
> 54
---------------+----------------------------------------------------------------
> --
PE             |
log_totallyco  |   .9089508   .4336923    -0.20   0.841     .3567814     2.315
> 68
               |
           par |
  Primiparous  |   .1095882    .104187    -2.33   0.020     .0170026    .70633
> 76
  Multiparous  |   .0454592   .0433697    -3.24   0.001     .0070072    .29491
> 66
               |
           bmi |   1.108368    .053704     2.12   0.034     1.007954    1.2187
> 86
         _cons |   .0817918   .2625357    -0.78   0.435     .0001515    44.146
> 77
---------------+----------------------------------------------------------------
> --
Note: _cons estimates baseline relative risk for each outcome.

196 . mlogit htncategoryaccaha log_acarot i.par bmi, vce(robust) rrr

Iteration 0:  Log pseudolikelihood = -226.42425
Iteration 1:  Log pseudolikelihood = -225.37838
Iteration 2:  Log pseudolikelihood = -221.11142
Iteration 3:  Log pseudolikelihood = -217.38583
Iteration 4:  Log pseudolikelihood = -215.95154
Iteration 5:  Log pseudolikelihood = -215.86289
Iteration 6:  Log pseudolikelihood = -215.86268
Iteration 7:  Log pseudolikelihood = -215.86268
```

```
Multinomial logistic regression                         Number of obs =    21
> 0
                                                        Wald chi2(12) =  27.5
> 1
                                                        Prob > chi2   = 0.006
> 5
Log pseudolikelihood = -215.86268                       Pseudo R2     = 0.046
> 6

─────────────┬──────────────────────────────────────────────────────────────
> ─
             │               Robust
htncategor~a │        RRR   std. err.      z    P>|z|     [95% conf. interval
> ]
─────────────┼──────────────────────────────────────────────────────────────
> ─
NT           │  (base outcome)
─────────────┼──────────────────────────────────────────────────────────────
> ─
CH           │
  log_acarot │   1.142157   .2540992     0.60   0.550     .7385104    1.76642
> 6
             │
         par │
Primiparous  │   .5637285   .5327791    -0.61   0.544     .0884302    3.59368
> 1
Multiparous  │   .4079897   .3773481    -0.97   0.332      .066585    2.49989
> 7
             │
         bmi │   .9615467   .0387673    -0.97   0.331     .8884888    1.04061
> 2
       _cons │   .8490826   1.421747    -0.10   0.922       .03189    22.6071
> 3
─────────────┼──────────────────────────────────────────────────────────────
> ─
GH           │
  log_acarot │   1.304226   .2193274     1.58   0.114     .9380137    1.81341
> 3
             │
         par │
Primiparous  │   1.660974    1.98035     0.43   0.670     .1605058    17.1883
> 9
Multiparous  │   1.226479   1.456058     0.17   0.863     .1197086    12.5659
> 3
             │
         bmi │   1.035968    .029426     1.24   0.213     .9798706    1.09527
```

```
> 8
       _cons |   .0395492   .0627773    -2.04   0.042      .001762    .887703
> 8
-------------+----------------------------------------------------------------
> -
PE           |
  log_acarot |   .6470416   .2210126    -1.27   0.202     .3312713    1.26380
> 7
             |
         par |
Primiparous  |    .144412   .1308543    -2.14   0.033      .024452    .852888
> 1
Multiparous  |   .0560245   .0551228    -2.93   0.003     .0081447    .385370
> 8
             |
         bmi |   1.096312   .0614623     1.64   0.101       .98223    1.22364
> 3
       _cons |   .2229863     .52827    -0.63   0.526     .0021465    23.1650
> 9
-------------+----------------------------------------------------------------
> -
Note: _cons estimates baseline relative risk for each outcome.
```

197 . mlogit htncategoryaccaha log_transbcarot i.par bmi, vce(robust) rrr

```
Iteration 0:  Log pseudolikelihood = -226.42425
Iteration 1:  Log pseudolikelihood = -223.52364
Iteration 2:  Log pseudolikelihood = -217.08645
Iteration 3:  Log pseudolikelihood = -216.55023
Iteration 4:  Log pseudolikelihood = -213.42046
Iteration 5:  Log pseudolikelihood = -213.28119
Iteration 6:  Log pseudolikelihood = -213.28072
Iteration 7:  Log pseudolikelihood = -213.28072

Multinomial logistic regression                         Number of obs =    21
> 0
                                                        Wald chi2(12) =  35.5
> 1
                                                        Prob > chi2   = 0.000
> 4
Log pseudolikelihood = -213.28072                       Pseudo R2     = 0.058
> 0
```

```
─────────────────┬──────────────────────────────────────────────────────────────
> ─────
                 │              Robust
htncategoryac~a  │        RRR   std. err.      z    P>|z|     [95% conf. inter
> val]
─────────────────┼──────────────────────────────────────────────────────────────
> ─────
NT               │  (base outcome)
─────────────────┼──────────────────────────────────────────────────────────────
> ─────
CH               │
log_transbcarot  │   1.217462   .3026995     0.79   0.429     .7478593    1.98
> 1942
                 │
             par │
   Primiparous   │    .587024   .5544754    -0.56   0.573      .092183    3.73
> 8186
   Multiparous   │   .4308401   .3992398    -0.91   0.364     .0700723    2.64
> 9022
                 │
             bmi │   .9639081   .0371868    -0.95   0.341     .8937106    1.03
> 9619
           _cons │   .4547325    .854543    -0.42   0.675      .011433    18.0
> 8639
─────────────────┼──────────────────────────────────────────────────────────────
> ─────
GH               │
log_transbcarot  │   1.220737   .2293955     1.06   0.289     .8446328    1.76
> 4315
                 │
             par │
   Primiparous   │   1.839848   2.170181     0.52   0.605     .1822859    18.5
> 6996
   Multiparous   │   1.376586   1.617651     0.27   0.786     .1375756    13.7
> 7417
                 │
             bmi │   1.032542   .0291293     1.14   0.256     .9769989    1.09
> 1242
           _cons │   .0385545   .0689608    -1.82   0.069     .0011576     1.2
> 8405
─────────────────┼──────────────────────────────────────────────────────────────
> ─────
PE               │
log_transbcarot  │   .3461519   .1039454    -3.53   0.000      .192159    .623
> 5521
                 │
```

```
             par │
   Primiparous  │   .1298412   .1132564    -2.34   0.019     .0234929    .717
> 6105
   Multiparous  │    .036992   .0340348    -3.58   0.000     .0060947     .22
> 4524
                │
             bmi│   1.074608   .0603464     1.28   0.200     .9626077    1.19
> 9639
           _cons│   15.41606   39.20706     1.08   0.282     .1054743    2253
> .204
────────────────┴──────────────────────────────────────────────────────────────
> ─────
Note: _cons estimates baseline relative risk for each outcome.
```

198 . mlogit htncategoryaccaha log_cisbcarot i.par bmi, vce(robust) rrr

```
Iteration 0:  Log pseudolikelihood = -225.33114
Iteration 1:  Log pseudolikelihood = -223.68322
Iteration 2:  Log pseudolikelihood = -217.80442
Iteration 3:  Log pseudolikelihood = -217.41238
Iteration 4:  Log pseudolikelihood = -214.39724
Iteration 5:  Log pseudolikelihood = -214.23237
Iteration 6:  Log pseudolikelihood =  -214.2317
Iteration 7:  Log pseudolikelihood =  -214.2317

Multinomial logistic regression                         Number of obs =    20
> 8
                                                        Wald chi2(12) =  37.7
> 8
                                                        Prob > chi2   = 0.000
> 2
Log pseudolikelihood = -214.2317                        Pseudo R2     = 0.049
> 3

──────────────┬──────────────────────────────────────────────────────────────
> ──
              │               Robust
htncategory~a │        RRR   std. err.      z    P>|z|     [95% conf. interva
> l]
──────────────┼──────────────────────────────────────────────────────────────
> ──
NT            │  (base outcome)
──────────────┼──────────────────────────────────────────────────────────────
> ──
CH            │
log_cisbcarot │    1.09852   .3113265     0.33   0.740     .6303372    1.9144
```

```
> 46
                |
            par |
   Primiparous  |   .5918105   .5563758    -0.56   0.577     .0937447      3.73
> 61
   Multiparous  |   .4356886   .4019318    -0.90   0.368     .0714373    2.6572
> 19
                |
            bmi |   .9617221   .0378234    -0.99   0.321     .8903748    1.0387
> 87
          _cons |   1.043921   1.659775     0.03   0.978     .0462706    23.552
> 11
----------------+---------------------------------------------------------------
> --
GH              |
log_cisbcarot   |   1.196419   .2341813     0.92   0.360     .8152173    1.7558
> 75
                |
            par |
   Primiparous  |   1.810329   2.143453     0.50   0.616     .1777967     18.43
> 28
   Multiparous  |   1.363745   1.608986     0.26   0.793     .1350396    13.772
> 27
                |
            bmi |   1.034134   .0292388     1.19   0.235     .9783862    1.0930
> 59
          _cons |   .0657414   .1027651    -1.74   0.082     .0030709    1.4073
> 88
----------------+---------------------------------------------------------------
> --
PE              |
log_cisbcarot   |   .4160421   .1834499    -1.99   0.047     .1753104      .987
> 34
                |
            par |
   Primiparous  |   .1468313    .136471    -2.06   0.039     .0237507    .90773
> 76
   Multiparous  |   .0552602   .0534352    -2.99   0.003     .0083047    .36770
> 69
                |
            bmi |    1.07212   .0676421     1.10   0.270     .9474132    1.2132
> 42
          _cons |   .7008404   1.836888    -0.14   0.892     .0041175    119.28
> 91
----------------+---------------------------------------------------------------
> --
```

```
Note: _cons estimates baseline relative risk for each outcome.

199 . mlogit htncategoryaccaha log_totalbcarot i.par bmi, vce(robust) rrr

Iteration 0:  Log pseudolikelihood = -226.42425
Iteration 1:  Log pseudolikelihood = -223.59078
Iteration 2:  Log pseudolikelihood = -217.22922
Iteration 3:  Log pseudolikelihood = -216.80933
Iteration 4:  Log pseudolikelihood = -213.55396
Iteration 5:  Log pseudolikelihood =  -213.3922
Iteration 6:  Log pseudolikelihood = -213.39164
Iteration 7:  Log pseudolikelihood = -213.39164

Multinomial logistic regression                         Number of obs =    21
> 0
                                                        Wald chi2(12) =  35.7
> 2
                                                        Prob > chi2   = 0.000
> 4
Log pseudolikelihood = -213.39164                       Pseudo R2     = 0.057
> 6

────────────────┬──────────────────────────────────────────────────────────────
> ─────
                │                Robust
htncategoryac~a │        RRR   std. err.      z    P>|z|     [95% conf. inter
> val]
────────────────┼──────────────────────────────────────────────────────────────
> ─────
NT              │  (base outcome)
────────────────┼──────────────────────────────────────────────────────────────
> ─────
CH              │
log_totalbcarot │   1.213328   .3055325     0.77   0.443     .7406861     1.9
> 8757
                │
            par │
   Primiparous  │   .5870056   .5545694    -0.56   0.573     .0921458     3.7
> 3946
   Multiparous  │   .4303805   .3989436    -0.91   0.363     .0699563    2.64
> 7759
                │
            bmi │   .9637871     .03722    -0.96   0.340     .8935297    1.03
> 9569
          _cons │   .4576787   .8751892    -0.41   0.683     .0107861    19.4
> 2039
```

```
──────────────────┼──────────────────────────────────────────────────────────
> ─────
GH                │
log_totalbcarot   │   1.227447   .2316418     1.09   0.278     .8479402    1.77
> 6806
                  │
              par │
     Primiparous  │   1.836437   2.169206     0.51   0.607     .1813566    18.5
> 9596
     Multiparous  │   1.373625   1.616573     0.27   0.787       .13681    13.7
> 9173
                  │
              bmi │   1.032805   .0291536     1.14   0.253     .9772171    1.09
> 1555
            _cons │   .0366816   .0663316    -1.83   0.068     .0010598    1.26
> 9647
──────────────────┼──────────────────────────────────────────────────────────
> ─────
PE                │
log_totalbcarot   │   .3491726   .1070651    -3.43   0.001     .1914434    .636
> 8541
                  │
              par │
     Primiparous  │   .1314732   .1150157    -2.32   0.020     .0236694    .730
> 2769
     Multiparous  │   .0380066   .0349152    -3.56   0.000      .006279    .230
> 0521
                  │
              bmi │   1.074422    .060627     1.27   0.203     .9619306    1.20
> 0069
            _cons │   16.05818   42.04504     1.06   0.289     .0948419    2718
> .896
──────────────────┴──────────────────────────────────────────────────────────
> ─────
Note: _cons estimates baseline relative risk for each outcome.

200 .
    end of do-file

201 . log off
          name:  <unnamed>
           log:  /Users/colmanfreel/Downloads/Carotenoids in HDP.smcl
      log type:  smcl
     paused on:   8 Sep 2025, 22:49:04
─────────────────────────────────────────────────────────────────────────────
```
