# Supplementary figures and images for: Differential Plasma Carotenoid Profiles in Hypertensive Disorders of Pregnancy
Source: Nutrients. 2025 Sep 29;17(19):3104. doi: 10.3390/nu17193104 (PMC12525936; doi:10.3390/nu17193104)

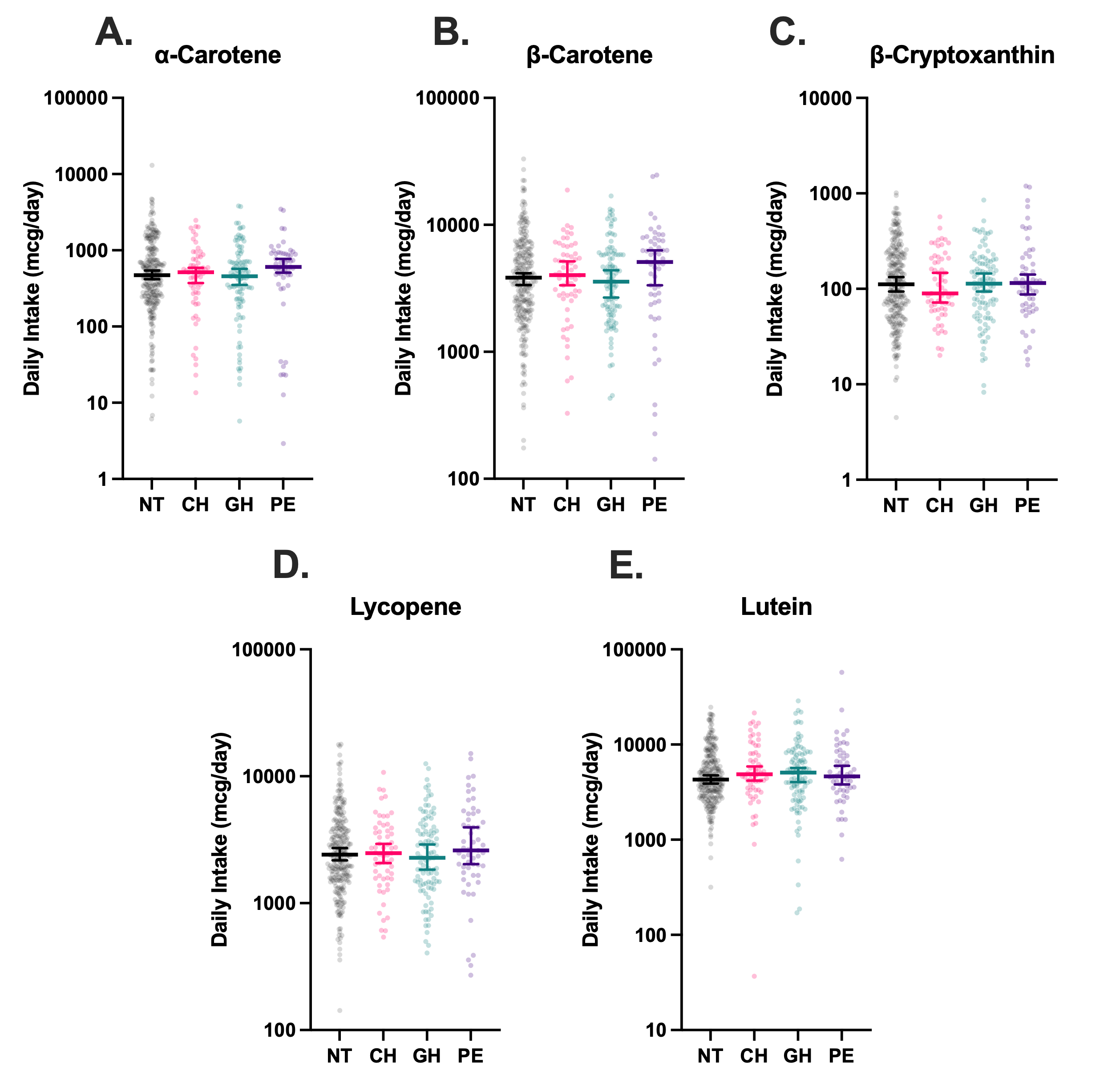

Supplement: Supplementary file 1 [file nutrients-17-03104-s001.zip › Supplementary Figures/Figure S1.png]

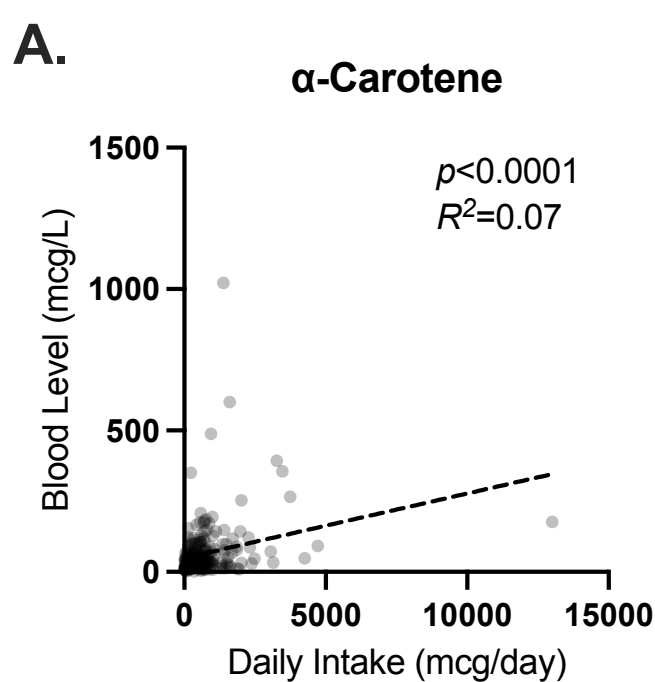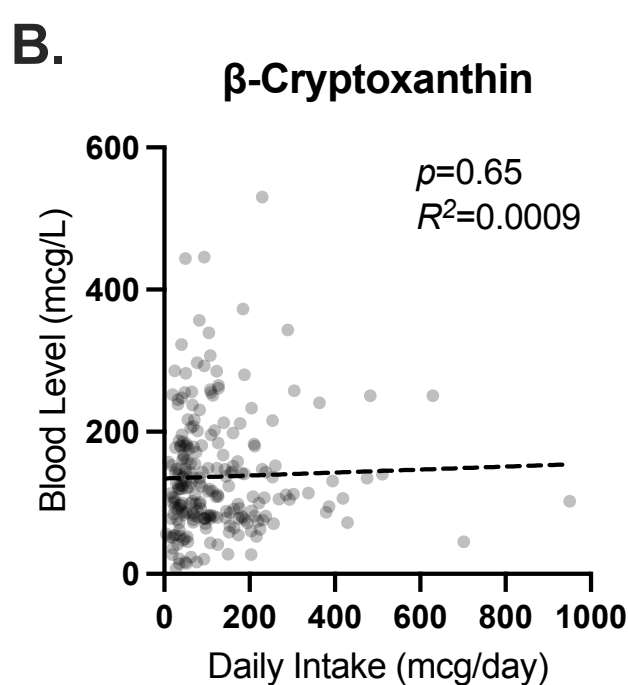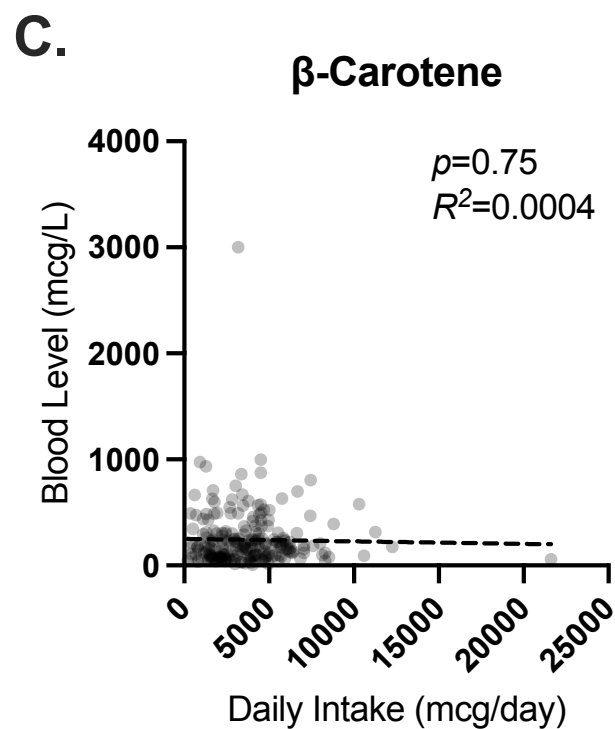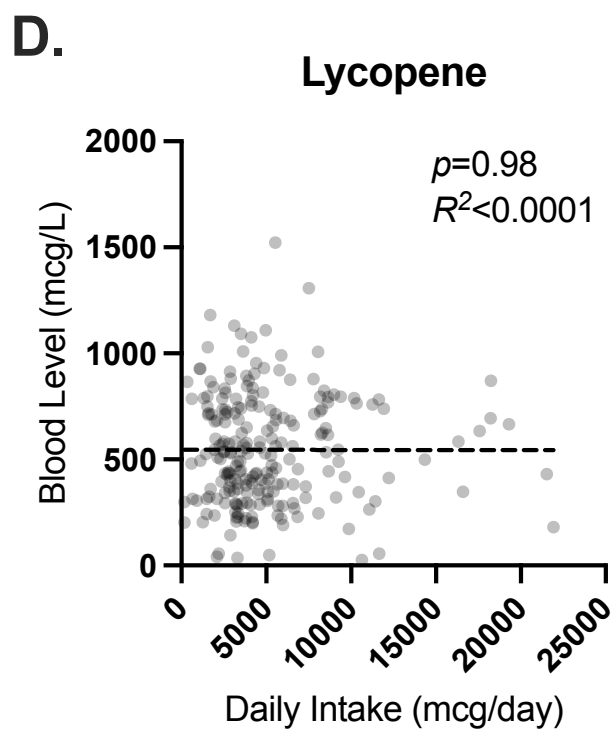

Supplement: Supplementary file 1 [file nutrients-17-03104-s001.zip › Supplementary Figures/Figure S2.pdf]
